# A comparative study of the digestion behavior and functionality of protein from chia (*Salvia hispanica* L.) ingredients and protein fractions

**DOI:** 10.1016/j.crfs.2024.100684

**Published:** 2024-01-24

**Authors:** Yan Wang, Alan Javier Hernández-Alvarez, Francisco M. Goycoolea, Cristina Martínez-Villaluenga

**Affiliations:** a School of Food Science & Nutrition, University of Leeds, LS2 9JT, Leeds, UK; b Department of Technological Processes and Biotechnology, Institute of Food Science, Technology and Nutrition (ICTAN-CSIC), Jose Antonio Novais 6, 28040, Madrid, Spain

**Keywords:** Anti-inflammatory, Antioxidant, Chia, *In vitro* digestion, Protein concentrate, Globulins

## Abstract

Protein derived from chia (*Salvia hispanica* L.), characterized by a balanced amino acid composition, represents a potentially healthier and environmentally friendly alternative poised for innovation within the plant-based food sector. It was hypothesized that the growing location of chia seeds and processing techniques used might influence protein digestion patterns, which in turn could affect the biological functions of the digestion products. To examine this hypothesis, we assessed the gastrointestinal fate of degummed-defatted flour (DDF), protein concentrate (PC), and isolated albumin (Alb) and globulin (Glo) fractions. Furthermore, we compared the antioxidant and anti-inflammatory activities of the resulting digesta by means of *in vitro* and cellular assays. Post-gastrointestinal digestion, the PC exhibited elevated levels of soluble protein (7.6 and 6.3 % for Mexican and British PC, respectively) and peptides (24.8 and 27.9 %, respectively) of larger molecular sizes compared to DDF, Alb, and Glo. This can be attributed to differences in the extraction/fractionation processes. Leucine was found to be the most prevalent amino acids in all chia digesta. Such variations in the digestive outcomes of chia protein components significantly influenced the bioactivity of the intestinal digestates. During gastrointestinal transit, British Glo exhibited the best reactive oxygen species (ROS) inhibition activity in oxidative-stressed RAW264.7 macrophages, while Mexican digesta outperformed British samples in terms of ROS inhibition within the oxidative-stressed Caco-2 cells. Additionally, both Mexican and British Alb showed effectively anti-inflammatory potential, with keratinocyte chemoattractant (KC) inhibition rate of 82 and 91 %, respectively. Additionally, Mexican PC and Alb generally demonstrated an enhanced capacity to mitigate oxidative stress and inflammatory conditions *in vitro*. These findings highlight the substantial potential of chia seeds as functional food ingredients, resonating with the shifting preferences of health-conscious consumers.

## Highlights

• Chia protein concentrates showed elevated peptide content post-digestion.
• Protein concentrates and albumins showed the highest antioxidant activity.
• British chia globulins exhibited higher cellular reactive oxygen species inhibition.
• Mexican and British protein concentrates led in macrophages nitric oxide inhibition.
• Digested chia proteins fractions lowered IL-6 and KC pro-inflammatory cytokines.

## Graphical abstract

**Figure:**
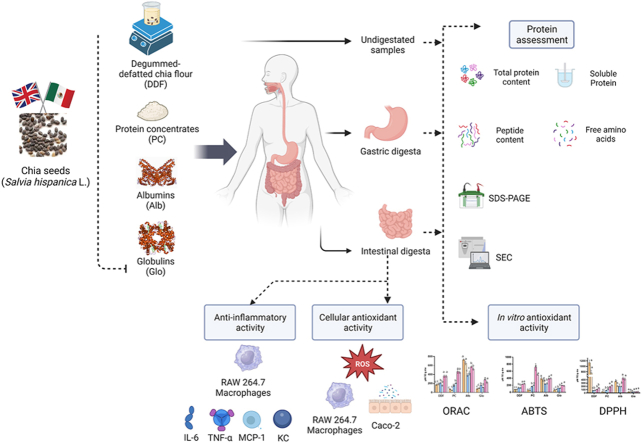

## 1 Introduction

Gastrointestinal digestion, a complex interplay of physical and chemical actions, is essential for breaking down food via various enzymes, thereby releasing nutrients for organismal absorption and utilization (Santos-Hernández et al., 2020). Ideally, the nutritional quality of foods, particularly its protein fraction, is best assessed through *in vivo* studies in humans or animals. However, these studies often present challenges such as high costs, technical complexities, time constraints, and ethical considerations (Dupont et al., 2019; Sousa et al., 2020). Consequently, the development of *in vitro* digestion models that accurately replicate human digestion processes has become a necessary and efficient alternative to *in vivo* experiments. Recognizing this need, a harmonized *in vitro* digestion protocol, reflecting human physiological conditions, was formulated by an international collaboration of scientists from over 35 countries, under the COST Action INFOGEST initiative (Brodkorb et al., 2019). The biological relevance and efficacy of the protocol has been validated for a wide range of proteins, particularly for milk, oat, sorghum, peanut and bean proteins through comparative studies with porcine and human digests (Egger et al., 2017; Sanchón et al., 2018; Sánchez-Velázquez et al., 2021; Sousa et al., 2020).

Chia (*Salvia hispanica* L.) seed, a pseudocereal grain originally from Southern Mexico and Northern Guatemala, has become a popular food due to its excellent nutritional composition consisting of 30–34 % dietary fiber, 26–41 % carbohydrates, and 29–39 % lipids rich in polyunsaturated fatty acids (Sánchez-Velázquez et al., 2023). As compared to most consumed cereal grains, chia seeds are characterized by a higher protein content (18–25 % dry weight, dw) and present a balanced amino acid composition that fulfills the dietary recommendations (Muñoz et al., 2012; FAO/WHO, 1991; FAO/WHO, 2011). Moreover, *in vitro* and *in silico* approaches have revealed that proteins from chia have a relevant role in health promotion through the release of bioactive peptides during digestion (Orona-Tamayo et al., 2015; Chim-Chi et al., 2018; Grancieri et al., 2019c; Martínez Leo and Segura Campos, 2020). In addition to macronutrients, chia seeds are a rich source of minerals (4–6 % dw, with 6 times more Ca than milk and 1.6 times more Fe than chickpea), vitamins (A, B, K, E, and D) and phytochemicals such as polyphenols and sterols (Cahill, 2003; Segura-Campos et al., 2014; Ullah et al., 2016; Coorey et al., 2012; Gu et al., 2021). Among polyphenols, phenolic acids (e.g., caffeic, chlorogenic, ferulic and rosmarinic acids) and flavanols (e.g., myricetin, quercetin and kaempferol) are particularly abundant in chia seeds. The outstanding nutritional composition of chia seeds have positioned this pseudocereal as a healthier and added-value alternative for food innovation considering the new consumer preferences for low-sugar, gluten-free alternatives, high fiber and protein, or mineral-enriched products, among others (Mesías et al., 2023). However, it is important to highlight that chia nutritional composition can vary based on external factors like climate, geographical location, soil characteristics, and year of cultivation (Grancieri et al., 2019a). Ayerza (2009) specifically demonstrated that factors such as temperature, climate, and altitude significantly influence the protein and oil content and fatty acid composition in chia seeds. This variability underlines the importance of considering environmental variables when evaluating the nutritional quality of chia seeds from different locations.

Generally, chia flour is obtained from seeds after partial oil extraction, resulting in a fiber-protein rich ingredient, suitable to enhance the nutritional properties of several food products (Aranibar et al., 2018; Mas et al., 2020). Extraction and fractionation processes are often applied to remove chia mucilage and produce protein concentrates from defatted chia flour (Zettel and Hitzmann, 2018). Degumming is performed to extract and/or remove chia mucilage by different procedures. Chia mucilage is a hydrocolloid with interesting technological properties used in the food industry as thickener, emulsifier, gelling agent, etc (Zettel and Hitzmann, 2018). Food processing of defatted and degummed chia flour (DDF) may continue with the wet protein extraction and isoelectric precipitation to yield chia protein concentrates (PC) and/or protein isolates that could find different applications in the food industry to develop plant-based foods.

Plant-based foods are generally lacking in certain essential amino acids and have lower digestibility due to the presence of antinutritional factors, thus, consequently regarded as of lower quality as compared to conventional sources of animal protein (Tachie et al., 2023). In a previous study, we studied the effects of defatting, degumming, and protein extraction/fractionation of chia flour on protein quality (Wang et al., 2023). The outcomes of the study demonstrated that defatting and degumming of chia flour followed by further wet protein extraction and isoelectric precipitation increased *in vitro* protein digestibility corrected amino acid score (IVPDCAAS) and reduced the concentration of certain antinutrients (phenolics and phytic acid) in PC as compared to DDF. In this work, it was hypothesized that the differences in the protein content and composition between chia seeds grown in different locations and chia seed processing can affect the protein digestion pattern and ultimately the bioavailability and biological functions of chia protein digestion products. To test this hypothesis, DDF, PC, albumin (Alb) and globulin (Glo) fractions were prepared from chia flours from two different locations (United Kingdom and Mexico). Samples were digested using the harmonized static INFOGEST 2.0 method (Brodkorb et al., 2019). The objective of this study was to: 1) assess and compare the protein digestion patterns of defatted chia seed fractions (DDF), protein concentrates (PC), albumins (Alb), and globulins (Glo) derived from British and Mexican chia seeds; 2) establish how the distribution of protein digestion products correlates with *in vitro* antioxidant activity through biochemical and cellular assays; and 3) assess the anti-inflammatory properties of the gastrointestinal digestates by evaluating the inhibition of nitric oxide (NO), interleukin-6 (IL-6), keratinocyte chemoattractant (KC), monocyte chemoattractant protein-1 (MCP-1), and tumor necrosis factor-alpha (TNF-α). Accomplishing these objectives will significantly contribute to the existing knowledge on chia seed protein digestibility and aims to provide crucial insights into the impact of chia protein digestion on health-promoting properties. This understanding is essential for advancing the application of chia seeds in health-centric dietary solutions and innovative food products.

## 2 Materials and methods

### 2.1 Preparation of chia ingredients and protein fractions

Chia seeds (*Salvia hispanica* L.) were obtained from United Kingdom (British seeds grown at Great Tey in Essex in 2019, Hodmedod's company, Essex, UK) and Mexico (provided by producers located in Guadalajara, Mexico, harvest of January 2019). Degummed-defatted chia flour (DDF), protein concentrates (PC), albumin (Alb) and globulin (Glo) fractions were prepared as described in a previous study (Wang et al., 2023). Briefly, the mucilage from the samples was removed by sonication at 50 % amplitude, 750 W for 4 min in an ultrasonic bath (Sonics, Tacoma, WA, USA), and manually separated from the seeds with the aid of a sieve (200 mm/30 mesh). The degummed seeds were ground using a commercial coffee grinder (De’Longhi KG200, Treviso, Italy) and the oil was extracted using hexane (1:5, w:v) under constant stirring for 2 h. The slurry was centrifuged (4816×*g*, 20 min, 4 °C) and DDF was left overnight under the fume cupboard and stored at 4 °C in vacuum-sealed bags. PC were produced from DDF by alkaline solubilization (pH 10) coupled to isoelectric precipitation (pH 4.5) and final centrifugation at 8288×*g*, 15 min at 4 °C (Avanti J-30I, Beckman Coulter, Brea, CA, USA). For Alb and Glo fractionation, DDF was dispersed in distilled water (1:40, w/v), stirred for 1 h at 4 °C and centrifuged at 13,000×*g* for 20 min at 4 °C. Supernatant (Alb fraction) was collected and freeze-dried (Labconco, Kansas, MO, USA), ground and stored at 4 °C in vacuum-sealed bags. The pellet was resuspended in 50 mM Tris containing 0.4 M NaCl at pH 8.0 (1:10, w/v), stirred and centrifuged as above. The supernatant (Glo fraction) was collected and freeze-dried, ground, and stored at 4 °C in vacuum-sealed bags.

### 2.2 *In vitro* simulated gastrointestinal digestion (SGID)

Chia samples were digested using a two-phase gastro-intestinal *in vitro* digestion model following a modified consensus INFOGEST 2.0 protocol (Brodkorb et al., 2019) with minor modifications. Briefly, all samples were mixed with a pre-warmed simulated salivary fluid (SSF) at a final ratio of 1:1 (w/v), intentionally excluding human salivary α-amylase due to the predominantly protein content in the samples studied. Immediately, the mixture was combined with simulated gastric fluid (SGF) and freshly prepared porcine pepsin from gastric mucosa (E.C. 3.4.23.1, Merck, Darmstadt, Germany) to a final ratio of food to SGF of 1:1 (v/v) and enzyme activity of 2000 U/mL, respectively. The pH of the mixture was adjusted to 3 with 1 M HCl before the gastric digestion was simulated by incubating sample tubes at 37 °C for 120 min. Thereafter, the gastric chyme was mixed with warmed simulated intestinal fluid (SIF) in a final ratio of 1:1 (v/v) to simulate the intestinal phase, respectively. Fresh porcine bile extract and pancreatin from porcine pancreas (Merck, Darmstadt, Germany) solution were added by considering the final concentration of 2.5 mM and trypsin enzymatic activity of 100 U/mL, respectively. The chyme pH was adjusted to 7 with 1 M NaOH and incubated at 37 °C for 120 min to complete 240 min *in vitro* digestion. The intestinal phase was stopped by heating in a boiling water bath for 5 min and immediately cooled in ice water. The digestates from both gastric (GP) and intestinal (IP) phases were stored at −80 °C and then freeze-dried (Labconco, Kansas City, MO, USA), ground and stored at 4 °C in vacuum-sealed bags until use.

### 2.3 Total protein content

The total protein content of protein samples was measured by the Dumas combustion method (AOAC, 1995) using the Trumac nitrogen analyzer (Leco Corporation, St Joseph, MA, USA). A conversion factor of 6.25 was used to convert nitrogen values to protein content. Results were expressed as g protein/100 g dw.

### 2.4 Soluble protein, peptides, and free amino acids analysis

Chia samples were dispersed at a final concentration of 1 % (w/v) in 2 mL Milli-Q water. Flour dispersions were stirred on thermomixer C (Thermo Fisher Scientific, Waltham, MA, USA) for 1 h and centrifuged (Eppendorf 5424 R, Thermo Fisher Scientific, Waltham, MA, USA) at 10,000×*g* and 4 °C for 5 min. The supernatant was collected, and protein concentration was measured using the Pierce 660 nm protein assay (Thermo Fisher Scientific, Waltham, MA, USA) according to the manufacturer's instructions. Bovine serum albumin (Merck, Darmstadt, Germany) was used as standard. Samples were measured in a Synergy HT microplate (BioTek Instruments, Winooski, VT, USA) at 660 nm.

Total peptide content was measured by Pierce Quantitative Colorimetric Peptide Assay Kit (Thermo Fisher Scientific, Waltham, MA, USA) in filtrates obtained from supernatant ultrafiltration using 10 kDa molecular weight (MW) cut-off membranes (Thermo Fisher Scientific, Waltham, MA, USA). Absorbance was read at 480 nm using a Synergy HT microplate reader (BioTek Instruments, Winooski, VT, USA). The results were expressed as g/100 g dw.

For extraction of free amino acids, 200 mg from each sample were taken and homogenized in 2 mL of 0.01 M HCl solution containing 10 μmol/mL norvaline as internal standard. This suspension was then centrifuged at 2500×*g* for 15 min and the supernatant was collected for subsequent analysis by reversed-phase high-performance liquid chromatography (RP-HPLC) and diode array detection (DAD) using an Agilent 1200 chromatographic system (Agilent Technologies, Inc., Wilmington, DE, USA) equipped with an G1329A automatic sampler. Separation of amino acids was performed into an Agilent Zorbax Eclipse Plus C18 column (4.6 × 250 mm, with particle size of 5 μm) at 40 °C. Two solvents (A and B) were used as the mobile phase. Solvent A consisted of 10 mM Na_2_HPO_4_:10 mM Na_2_B_4_O_7_, pH 8.2:5 mM NaN_3_ and solvent B was acetonitrile:methanol:water (45:45:10, v:v:v). The injection volume was 20 μL and the mobile phase flow rate was 1.5 mL/min. The gradient flow for chromatographic separation started from 2 % B for 0.5 min followed by 57 % B for 30 min then 100 % B for 10 min. Initial chromatographic conditions were set out for column re-equilibration between sample injections. Amino acid detection was performed using automated derivatization in the autosampler. Derivatization reagents: Borate buffers (0.4 M in water, pH 10.2), *o*-phthaldialdehyde (OPA, 10 mg/mL in 0.4 M borate buffer and 3-mercaptopropionic acid) and fluorenylmethoxycarbonyl (FMOC, 2.5 mg/mL in acetonitrile) were ready-made solutions supplied by Agilent. They were transferred from their container into an autosampler vial. DAD was set up for collecting two channels (Signal A 338 nm, to detect OPA derivatized amino acids and Signal B 262 nm, to detect FMOC-lys derivatized amino acids). Peak identification was performed by retention time comparison with amino acid standards. Standard solutions of 20 amino acids available from Agilent (1 nmol/μL) were used to prepare calibration curves. Calibration curves with standard concentration range from 10 to 1000 nmol/mL of individual free amino acids were determined, with each concentration measured in triplicate. The linearity was evaluated by the calibration curves for each standard and least-squares regression lines relating the absorbance peak area.

### 2.5 Sodium dodecyl sulfate-polyacrylamide gel electrophoresis (SDS-PAGE)

To assess the distribution of polypeptides in chia samples as well as in their corresponding gastric digestates, SDS-PAGE was performed using reagents and equipment purchased from Lifescience Technologies (Thermo Fisher Scientific, Waltham, MA, USA). Briefly, 10 μL of supernatant (section 2.4.) were mixed with 20 μL NuPAGE® lithium dodecyl sulfate (LDS) sample buffer (without *β*-mercaptoethanol), pH 8.4, making a total volume of 30 μL. Samples were heated at 70 °C for 10 min using a thermomixer C (Thermo Fisher Scientific, Waltham, MA, USA) and then centrifuged at 4 °C and 10,000×*g* for 5 min (Eppendorf 5424 R, Thermo Fisher Scientific, Waltham, MA, USA). For analysis, 13 μg protein/well and 5 μL of Novex® Sharp Prestained Protein Standard were loaded onto a 1.0 mm x 10 well 4–12 % gradient Bis-Tris gel. The protein separation was run in a Mini Gel Tank at 200 V for 35 min using NuPAGE® 2-(N-morpholino) ethanesulfonic acid-SDS running buffer. For the albumin fraction with low MW protein fragments, aliquots of 10 μL were mixed in 20 μL Tricine SDS sample buffer (1X). For analysis, 13 μg peptide/well and 5 μL of PageRuler Unstained Low Range Protein Ladder Standard onto 1.0 mm x 10 well Novex 16 % Tricine Gels. Protein separation was performed at 125 V for 90 min with Tricine SDS running buffer. Bands were stained using SimplyBlue and images were analyzed using ChemiDoc XRS+ and Quantity One software (Bio-Rad, Hercules, CA, USA).

### 2.6 Size exclusion chromatography

To assess changes in peptide size distribution of chia samples during gastric and intestinal phases, supernatants (section 2.4.) of sample suspensions, were analyzed by size exclusion chromatography as described by Rieder et al. (2021) with modifications. A solvent made up of 30 % acetonitrile and 0.05 % trifluoracetic acid (TFA) in water was used as the mobile phase. Supernatants (section 2.4.) were diluted 1:5 with mobile phase and filtered using a syringe filter (0.45 μm polyvinylidene difluoride (PVDF) membrane). Sample volumes of 10 μL were injected in HPLC-DAD (Waters, Milford, MA, USA) controlled by Empower (Waters, Milford, MA, USA) and eluted at 0.5 μL/min. Peptides were separated using a TSKgel column G2000SWXL (7600 × 7.5 mm; Tosoh Bioscience GmbH, Stuttgart, Germany) over 30 min and detected at a wavelength of 214 nm. Peptide MW was estimated based on elution time of a standard peptide mixture that included angiotensin II (1046 Da), met-enkephalin (573.611 Da), Leu-enkephalin (555.6 Da), Val-Tyr-Val (379.5 Da) and Gly-Tyr (235.24 Da).

### 2.7 *In vitro* antioxidant activity

Antioxidant activity was determined in flours before and after gastric and intestinal digestion using four methods: Oxygen Radical Absorbance Capacity (ORAC), 2,2-azino-bis-3-ethylbenzothiazoline-6-sulfonic acid (ABTS) radical scavenging assay, and 2,2-diphenyl-1-picrylhydrazyl (DPPH) radical scavenging assay and inhibition of intracellular reactive oxygen species (ROS) in *tert*-butyl hydroperoxide (*t*-BHP) challenged cell lines.

The ORAC method was executed according to Bautista-Expósito et al. (2021). The fluorescence of the samples was assessed using a battery of dilutions prepared in 75 mM sodium phosphate buffer (pH 7.4). The measurements were taken at 485 and 520 nm every 2 min for 2.5 h, using a Synergy HT microplate reader (BioTek Instruments, Winooski, VT, USA). Data were obtained using a Trolox standard curve (0–160 μM) and results were expressed as μM Trolox equivalents (TE)/g of sample.

The ABTS analysis was performed as described by (Martín-Diana et al., 2021). A series of sample dilutions were prepared in 0.1 M phosphate buffer with 0.15 M NaCl. Absorbances were measured at 734 nm every minute for 30 min, using the microplate reader. Data were obtained from a Trolox calibration curve (0–800 μM) and results were expressed as μM TE/g of sample.

The DPPH assay was conducted as previously reported by Brand-Williams et al. (1995). In this case, various dilutions of sample were prepared using Milli-Q water and absorbance was measured at 515 nm after 30 min of incubation. Data were obtained from a Trolox standard curve (0–200 μM) and results were expressed as μM TE/g of sample.

Cellular antioxidant activity was determined in two cell lines: murine RAW 264.7 macrophages (ATCC, TIB-71, Rockville, MD, USA) and human adenocarcinoma Caco-2 cells (ATCC, Rockville, MD, USA) cultured in Dulbecco's modified Eagle's Medium (DMEM) and Minimum Essential Medium Alpha (MEM-α), respectively, both containing 10 % fetal bovine serum (FBS) and 1 % penicillin-streptomycin (P/S). Cells were maintained in an oven incubator with a 5 % CO_2_ atmosphere at 37 °C. For experiments, cells were seeded into black 96-well plates (5 × 10^4^ cells/well) and allowed to attach overnight at 37 °C with 5 % CO_2_. Cells were treated with intestinal digests (0.5 and 3 mg/mL) previously dissolved in complete DMEM with 0.1 % of FBS and filtered using sterile filters of 0.22 μm (Sarstedt AG & Co KG, Nümbrecht, Germany). Treated cells were incubated at 37 °C with 5 % CO_2_ for 18 h, washed twice with 100 μL of ice-cold phosphate buffer saline (PBS) and incubated for 30 min at 37 °C and 5 % CO_2_ in darkness with 150 μL of 10 μM dichloro-dihydro-fluoresceine diacetate (H_2_DCFDA) in PBS. After incubation, cells were washed again with PBS followed by treatment with 200 μL of 2.5 mM *terc*-butylhydroperoxide (tBHP). Fluorescence intensity was recorded at λ_exc_ = 485 nm and λ_em_ = 535 nm wavelengths on a Synergy HT microplate reader (BioTek Instruments Inc., Winooski, VT, USA). Results were expressed as percentages concerning untreated cells (tBHP-). Data represents the mean and the standard deviation of eight biological replicates.

### 2.8 *In vitro* anti-inflammatory activity

Murine RAW264.7 macrophages were cultured in complete DMEM supplemented by 10 % FBS and 1 % P/S in a humidified incubator at 37 °C and 5 % CO_2_. Cells were seeded in 96-well plates at a density of 2.5 × 10^4^ cells/well. After overnight attachment, the cells were treated with intestinal digests (0.5 and 3 mg/mL complete growth medium) for 1 h and challenged with 1 μg/mL of lipopolysaccharide (LPS) for 23 h. After incubation, the cell spent media was collected for determination of nitric oxide (NO) via Griess reagent assay (Martinez-Villaluenga et al., 2009) and cytokine/chemokine (IL-6, KC, MCP-1, and TNF-α) were determined by flow cytometry using the Mouse Cytokine Magnetic kit (MCYTOMAG-70 K, Merck KGaA, Darmstadt, Germany). Analysis was performed on a Luminex XYP flow cytometer (Luminex Co., Austin, TX, USA) using the Belysa™ Data Analysis Software (version 1.2, Merck KGaA, Darmstadt, Germany). The spent medium was replaced with 100 μL of serum-free medium with CellTiter 96® Aqueous Non-Radioactive Cell Proliferation Assay solution (ratio 9:1, v/v) for 2 h at 37 °C in a humidified incubator with 5 % CO_2_. Absorbance was read at 490 nm on a Synergy HT microplate reader (Biotek Instruments, Winooski, VT, USA). Untreated RAW264.7 were considered as negative control (LPS−). Cell viability was expressed as a percentage of untreated cells (LPS−). Data represents the mean and the standard deviation of five biological replicates.

### 2.9 Statistical analysis

All the performed analyses were done in triplicates, except for the simulated digestion which was carried out in duplicates. The results were presented as mean ± standard deviation. Two-way ANOVA statistical tests (Tukey's HSD multiple comparison test) were performed on the collected data where appropriate with a 95 % confidence interval (GraphPad Prism v.9.0 software, Domatics, Stortford, UK). Statistical comparisons between two groups were determined by an unpaired two-tailed *t*-test. *P* < 0.05 was considered statistically significant for all comparisons.

## 3 Results and discussion

### 3.1 Total protein content in Mexican and British chia ingredients, Alb and Glo fractions

**Figure 1:**
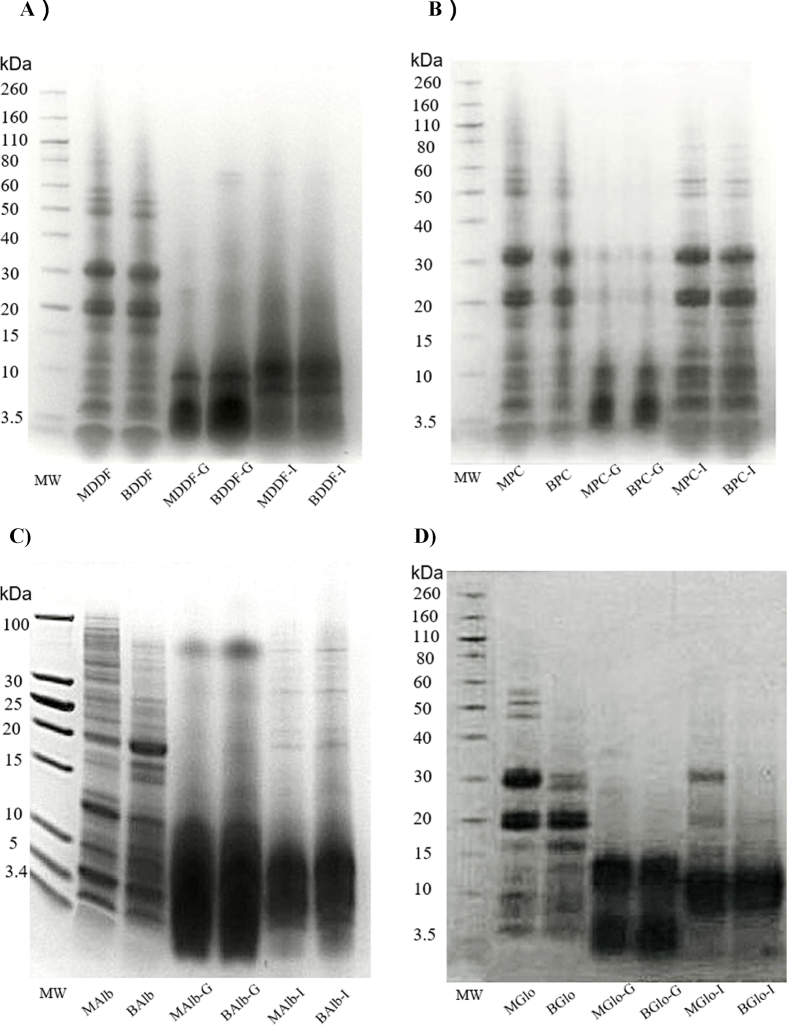
SDS-PAGE profiles of four different chia (*Salvia hispanica* L.) samples before and after gastric and intestinal digestion under non-reducing conditions: **A)** degummed-defatted flour; **B)** protein concentrates; **C)** albumin fraction; and **D)** globulin fraction. Abbreviations: MDDF, Mexican degummed-defatted flour; BDDF, British degummed-defatted flour; MPC, Mexican protein concentrates; BPC, British protein concentrates; MAlb, Mexican chia albumin; BAlb, British chia albumin; MGlo, Mexican chia globulin; BGlo, British chia globulin. G, gastric digestion endpoint; I, intestinal digestion endpoint. MW, molecular weight standard (kDa).

Total protein content of undigested chia DDF, PC, Alb, and Glo is presented in Fig. 1S (Supplementary Material). The total protein content in Mexican (MDDF) and British (BDDF) chia degummed and defatted flour was 35 and 37 g/100 g dw, respectively (data not shown). While, Mexican (MPC) and British (BPC) chia protein concentrates showed protein contents of 88 and 89 g/100 g dw, respectively, in line with previous reported values (Malik and Riar, 2022; Timilsena et al., 2016). The protein content in Alb fractions from MDDF and BDDF reached 38 and 57 g/100 g dw, respectively, whereas the protein content in the Glo fraction from MDDF and BDDF was 44 and 39 g/100 g dw, respectively. However, a previous study by Sandoval-Oliveros and Paredes-López (2013) determined the protein content in chia protein fractions from Colima, Mexico, finding 17 g/100 g dw in albumin and 52 g/100 g dw in globulin fractions. In contrast, Segura-Campos (2019) reported 21 g/100 g dw in albumin and 17 g/100 g dw in globulin fractions from chia seeds sourced from Yucatan, Mexico. These findings indicated that the origin of chia seeds influenced significantly (*p* < 0.05) the protein content of DDF, PC, Alb and Glo fractions.

### 3.2 Changes in soluble protein in Mexican and British chia ingredients, Alb and Glo fractions during gastrointestinal transit

Soluble protein (SP) content was measured during SGID to understand the variations in protein digestibility among chia ingredients and protein fractions. As shown in Table 1, prior to SGID and at neutral pH, MDDF and BDDF exhibited SP contents of 28 and 34 g/100 g dw, respectively. Among all samples, PC displayed the highest SP value, with 62 g/100 g dw for MPC and 75 g/100 g dw for BPC, in coherence to their higher protein content. The SP content in Mexican (MAlb) and British (BAlb) albumin was 32.5 and 56 g/100 g dw, respectively, while in Mexican (MGlo) and British (BGlo) globulin, it was 35 and 33 g/100 g dw, respectively, with statistically significant differences (*p* < 0.05) observed between locations.

**Table 1:** Soluble protein, peptides and free amino acids content (g/100 g dw) during gastrointestinal transit of ingredients and protein fractions prepared from British and Mexican chia (*Salvia hispanica* L.) seeds.

Chia samples	Location	Digestion phase	Soluble protein	Peptides	Free amino acids
DDF	M	U	27.64±0.09^a,E^	3.46±0.25^c,C^	1.20±0.01^b,C^
		G	4.09±0.08^b,A^	14.43±0.61^b,C^	1.22±0.06^b,C^
		I	1.55±0.08^b,B^	26.31±0.82^a,D^	3.72±0.12^a,AB^
	B	U	33.59±2.48^a,D^	3.11±0.06^c,C^	0.96±0.05^b,C^
		G	3.29±0.11^b,A^	16.56±1.55^b,C^	1.15±0.01^b,C^
		I	1.43±0.07^b,B^	27.10±1.88^a,D^	3.87±0.14^a,AB^
PC	M	U	62.44±0.21^a,B^	5.08±0.11^c,C^	0.42±0.01^b,C^
		G	6.17±0.07^b,A^	36.52±1.51^b,BC^	0.30±0.02^b,C^
		I	7.57±0.26^b,A^	44.34±2.16^a,B^	3.38±0.91^a,B^
	B	U	75.34±0.96^a,A^	5.62±0.30^c,C^	0.43±0.02^b,C^
		G	6.78±0.18^b,A^	32.56±1.93^b,C^	0.28±0.01^b,C^
		I	6.33±0.21^b,AB^	45.55±2.63^a,B^	3.50±0.09^a,AB^
Alb	M	U	32.54±0.70^a,D^	31.02±3.83^c,A^	5.06±0.69^a,A^
		G	6.001±0.18^b,A^	52.46±8.57^b,A^	4.02±0.05^b,B^
		I	6.16±0.15^b,AB^	60.09±1.17^a,A^	4.26±0.85^b,A^
	B	U	55.68±0.95^a,C^	24.08±3.96^b,B^	4.02±0.41^b,B^
		G	3.58±0.04^b,A^	39.60±2.06^a,B^	5.71±0.28^b,A^
		I	5.97±0.16^b,AB^	41.98±1.78^a,B^	4.04±0.61^a,AB^
Glo	M	U	35.46±0.47^a,D^	2.52±0.27^c,C^	0.64±0.01^b,C^
		G	8.69±0.11^b,A^	23.73±3.35^b,D^	0.59±0.03^b,C^
		I	3.30±0.11^c,AB^	32.47±2.38^a,C^	3.71±0.43^a,AB^
	B	U	33.26±0.24^a, D^	3.79±0.57^b,C^	0.74±0.01^b,C^
		G	6.05±0.11^b,A^	25.71±2.32^a,D^	0.69±0.04^b,C^
		I	1.56±0.03^b,AB^	29.21±2.37^a,CD^	4.06±0.63^a,AB^

Data are the mean ± standard deviation of three replicates. Different lowercase letter within the column indicates statistical differences among different digestion phases of the same chia sample (*p* < 0.05, Tukey test). Different uppercase letter within the column indicates statistical differences among chia samples at the same digestion phases (*p* < 0.05, Tukey test). Abbreviations: DDF, degummed-defatted chia flour; PC, protein concentrate; Alb, albumin; Glo, globulin; M, Mexican chia samples; B, British chia samples; U, undigested; G, endpoint of gastric digestion; I, endpoint of intestinal digestion.

At the end of gastric digestion, a pronounced reduction in SP content (over 80 %; *p* < 0.05) was observed in all samples. This finding can be attributed to changes in pH manifesting as an acidic milieu or fluctuations in ionic strength that occurred during food digestion. These alterations are recognized to impede the interactive dynamics between proteins and water, thereby eliciting an elevation in protein-protein interactions that ultimately induce protein aggregation and thus precipitation (Culbertson, 2005; Yuliana et al., 2014; Ivanova et al., 2013). Previous studies demonstrated that chia proteins have the isoelectric point at pH 3 in which protein solubility ranges between 5 and 10 % (Timilsena et al., 2016, Coelho and DE Las Mercedes Salas-Mellado, 2018). Among chia protein fractions, MGlo exhibited the highest SP content at 8.7 g/100 g dw, while BDDF showed the lowest content at 3.3 g/100 g dw. Similarly, Julio et al. (2019) showed a higher protein solubility of the Glo fraction from Mexican chia seeds at pH 3. This phenomenon may be attributed to the augmented presence of hydrophilic amino acid residues on the surface of Glo, facilitating interactions with water molecules and consequently enhancing solubility (Zeng et al., 2013).

At the endpoint of intestinal digestion, a substantial reduction of approximately 90 % in SP content was observed in all samples as compared to undigested counterparts (*p* < 0.05, Table 1). Although chia protein solubility increases at neutral pH values reached 35 % (Khushairay et al., 2023), lower soluble protein values at the end of intestinal digestion may be attributed to the extensive proteolysis caused by pancreatic proteases (e.g. trypsin and chymotrypsin) and peptidases producing smaller peptide fragments and free amino acids. Among the studied samples, the MPC and BPC displayed the highest SP content (7.6 g/100 g dw and 6.4 g/100 g dw, respectively), while MDDF and BDDF exhibited the lowest content (1.6 g/100 g dw and 1.4 g/100 g dw, respectively).

Chia processing (extraction/fractionation) affected the remaining SP content at the end of gastrointestinal digestion. Regardless of chia seed origin, PC and Alb fraction showed higher SP content in intestinal digesta than DDF and Glo fractions (Table 1, *p* < 0.05) suggesting a lower protein digestibility of the former. These results are coherent with the higher concentration of antinutrients (trypsin inhibitors and polyphenols) observed in PC and Alb fraction as compared to DDF and Glo fraction (Wang et al., 2023).

Notably, there was a significant influence of chia seeds origin on the content of SP at the beginning and the end of gastric and duodenal digestion (Table 1, *p* < 0.05). Generally, British chia samples exhibited higher SP content than Mexican samples before and at the end of digestion. These results indicated that proteins in ingredients prepared from British chia seeds have a higher resistance to digestion than those prepared from Mexican chia seeds. Therefore, the origin of chia seed may influence protein digestibility.

### 3.3 Changes in peptide content in Mexican and British chia ingredients, Alb and Glo fractions during gastrointestinal transit

Except for Alb fraction, peptide content in undigested chia samples was low varying between 2.5 and 5.6 g/100 g dw (Table 1). The lowest peptide content was observed in MGlo, followed by BDDF < MDDF < BGlo < MPC < BPC (*p* > 0.05, Table 1). Alb exhibited the highest peptide content before digestion, with values of 31 and 24 g/100 g dw in MAlb and BAlb, respectively (*p* < 0.05). This higher peptide content observed in Alb could potentially be attributed to the use of water as a solvent during extraction of Alb, as Alb are water soluble proteins with low MW (Orona-Tamayo et al., 2015, 2017). Water, being a polar solvent, possesses the ability to efficiently dissolve and interact with the polar and hydrophilic constituents of peptides (Wolfenden, 1978).

During SGID, peptide content increased rapidly in all samples (Table 1, *p* < 0.05) as a result of the action of digestive enzymes (pepsin and pancreatin) which selectively cleave peptide bonds, breaking down proteins into peptides. Regardless of chia seed samples, Alb followed by PC gastric and intestinal digestates showed a significantly higher peptide content as compared to DDF and Glo (*p* < 0.05). A higher content of digestion resistant peptides in PC and Alb gastric and intestinal digestates was indicative of a lower degree of proteolysis, in consistence with the higher concentration of antinutrients reported for these samples (Wang et al., 2023). Considering the origin of chia seeds, the peptide content of DDF and Alb derived from Mexican chia seeds was higher than British before SGID. However, after SGID, protein fractions (Alb and Glo) isolated from Mexican chia seeds exhibited higher peptide content indicating a higher digestibility, which was confirmed by a higher degree of hydrolysis (data not shown).

### 3.4 Changes in free amino acid content in Mexican and British chia ingredients, Alb and Glo fractions during gastrointestinal transit

FAA in undigested chia samples was low ranging from 0.42 to 5.06 g/100 g dw. Alb fraction showed the highest FAA content as compared to DDF, PC and Glo (Table 1, *p* < 0.05). Regarding chia seed origin, statistical differences were observed particularly in MAlb (5.06 g/100 g dw) that showed higher FAA levels than BAlb (4.02 g/100 g dw).

FAA content increased in a greater extent during intestinal digestion phase in all studied chia samples (Table 1, *p* < 0.05). At the end of gastrointestinal digestion, no statistical differences were observed in the FAA content when comparisons were performed by chia seed location or type of processing. Table 2 shows the FAA composition of undigested DDF, PC, Alb, and Glo and their corresponding digestates after *in vitro* gastric and intestinal digestion. The data analysis revealed that, in most cases, there were no significant differences (*p* < 0.05) between DDF and PC prior to SGID. This suggests that the technique used for PC extraction has minimal impact on the AA composition (Wang et al., 2023).

**Table 2:** Free amino acids measured in undigested chia (*Salvia hispanica* L.) ingredients and protein fractions and the digestates after *in vitro* gastric and intestinal digestion.

Amino acids	Location	Digestion Phase	Chia (*Salvia hispanica* L.) samples			
			DDF	PC	Alb	Glo
**Non-essential amino acids (g/100 g dw)**						
Asparagine	M	U	0.015±0^a,B^	0.0025±0.004^a,B^	0.105±0.030^ab,A^	0.008±0^a,B^
		G	0.019±0.001^a,B^	0.001±0^a,B^	0.086±0.002^b,A^	0.004±0^a,B^
		I	0.034±0^a,AB^	0.021±0.002^a,B^	0.043±0.011^c,A^	0.023±0.004^a,AB^
	B	U	0.015±0.001^a,B^	0.006±0^a,B^	0.084±0.013^b,A^	0.01±0^a,B^
		G	0.017±0.001^a,B^	0.002±0^a,B^	0.125±0.006^a,A^	0.006±0^a,B^
		I	0.032±0.001^a,A^	0.022±0^a,A^	0.035±0.006^c,A^	0.027±0.004^a,A^
Glutamic acid	M	U	0.102±0.001^a,B^	0.02±0^b,B^	0.763±0.132^a,A^	0.036±0.001^a,B^
		G	0.055±0.001^a,B^	0.017±0.001^b,B^	0.599±0.006^b,A^	0.031±0.001^a,B^
		I	0.127±0.002^a,B^	0.081±0.011^b,B^	0.296±0.071^c,A^	0.098±0.011^a,B^
	B	U	0.103±0.001^a,B^	0.017±0.001^b,B^	0.617±0.071^b,A^	0.048±0.001^a,B^
		G	0.057±0.001^a,B^	0.018±0.001^b,B^	0.848±0.145^a,A^	0.042±0.001^a,B^
		I	0.125±0.001^a,AB^	0.086±0.001^a,C^	0.219±0.037^c,A^	0.112±0.016^a,B^
Serine	M	U	0.046±0.001^b,B^	0.014±0^b,C^	0.234±0.021^a,A^	0.025±0.001^b,BC^
		G	0.045±0.001^b,B^	0.010±0.001^b,C^	0.170±0.004^b,A^	0.021±0.001^b,BC^
		I	0.123±0.001^a,B^	0.091±0.011^a,C^	0.162±0.033^b,A^	0.114±0.012^a,BC^
	B	U	0.038±0.003^b,B^	0.016±0.001^b,B^	0.182±0.011^b,A^	0.033±0^b,B^
		G	0.038±0.001^b,B^	0.009±0.001^b,C^	0.245±0.013^a,A^	0.030±0.001^b,BC^
		I	0.12±0.001^a,A^	0.090±0.001^a,B^	0.125±0.017^c,A^	0.125±0.015^a,A^
Glutamine	M	U	0.047±0.001^b,B^	0.016±0^b,B^	0.278±0.032^a,A^	0.021±0^b,B^
		G	0.049±0.001^b,B^	0.012±0.001^b,B^	0.150±0.001^c,A^	0.022±0.001^b,B^
		I	0.228±0.002^a,A^	0.207±0.009^a,A^	0.201±0.041^b,A^	0.237±0.023^a,A^
	B	U	0.038±0.001^b,B^	0.014±0.001^b,B^	0.192±0.015^bc,A^	0.025±0.001^b,B^
		G	0.039±0.001^b,B^	0.011±0.001^b,B^	0.237±0.008^ab,A^	0.025±0^b,B^
		I	0.242±0.001^a,A^	0.210±0.001^a,A^	0.174±0.024^bc,B^	0.238±0.033^a,A^
Glycine	M	U	0.023±0^b,B^	0.005±0.001^b,C^	0115±0.004^b,A^	0.009±0.001^b,BC^
		G	0.021±0.001^b,B^	0.003±0^b,C^	0.070±0.001^c,A^	0.007±0^b,BC^
		I	0.1±0.001^a,B^	0.076±0^a,C^	0.15±0.021^a,A^	0.091±0.006^a,B^
	B	U	0.021±0.001^b,B^	0.006±0^b,B^	0.075±0.003^c,A^	0.01±0^b,B^
		G	0.019±0.001^b,B^	0.003±0^b,C^	0.112±0.006^b,A^	0.008±0.001^b,BC^
		I	0.105±0.002^a,A^	0.083±0.001^a,B^	0.103±0.006^b,AB^	0.096±0.006^a,AB^
Arginine	M	U	0.138±0.002^b,B^	0.037±0.001^b,B^	0.398±0.034^b,A^	0.109±0.001^b,B^
		G	0.120±0.006^b,B^	0.027±0.001^b,B^	0.350±0.002^b,A^	0.105±0.004^b,B^
		I	0.722±0.004^a,A^	0.723±0.037^a,A^	0.554±0.113^ab,B^	0.772±0.008^a,A^
	B	U	0.114±0.006^b,B^	0.036±0^b,B^	0.351±0.023^b,A^	0.126±0.001^b,B^
		G	0.103±0.001^b,B^	0.026±0.001^b,B^	0.432±0.021^b,A^	0.119±0.002^b,B^
		I	0.755±0.003^a,B^	0.755±0.001^a,B^	0.631±0.087^a,B^	0.833±0.114^a,A^
Alanine	M	U	0.063±0^b,B^	0.049±0.001^c,B^	0.369±0.045^ab,A^	0.027±0.006^b,B^
		G	0.069±0.001^b,B^	0.014±0.001^b,B^	0.325±0.006^b,A^	0.028±0.001^b,B^
		I	0.142±0^a,B^	0.122±0.001^a,B^	0.238±0.044^c,A^	0.139±0.009^a,B^
	B	U	0.059±0.003^b,B^	0.070±0.002^c,B^	0.321±0.026^b,A^	0.042±0.001^b,B^
		G	0.065±0^b,B^	0.013±0.001^b,C^	0.409±0.021^a,A^	0.035±0.001^b,BC^
		I	0.144±0.001^a,AB^	0.114±0.006^a,B^	0.187±0.021^c,A^	0.151±0.032^a,AB^
Tyrosine	M	U	0.042±0.001^b,B^	0.013±0^b,B^	0.234±0.040^b,A^	0.031±0^b,B^
		G	0.063±0.002^b,B^	0.016±0.001^b,B^	0.271±0.002^b,A^	0.042±0.004^b,B^
		I	0.21±0.001^a,B^	0.168±0.011^a,B^	0.335±0.077^ab,A^	0.225±0.042^a,B^
	B	U	0.04±0.003^b,B^	0.013±0^b,B^	0.238±0.027^b,A^	0.045±0.001^b,B^
		G	0.055±0.001^b,B^	0.017±0.002^b,B^	0.302±0.009^b,A^	0.061±0^b,B^
		I	0.223±0.004^a,BC^	0.178±0.001^a,C^	0.406±0.074^a,A^	0.294±0.074^a,B^
Cysteine	M	U	0.007±0^bc,A^	0.005±0.002^b,A^	0.007±0.001^b,A^	0.004±0^b,A^
		G	0.015±0.002^bc,B^	0.021±0.001^a,AB^	0.019±0.006^b,AB^	0.029±0.001^a,A^
		I	0.064±0.004^a,A^	0.018±0.001^ab,BC^	0.014±0.01^b,C^	0.028±0^a,B^
	B	U	0.003±0^c,B^	0.006±0^b,AB^	0.018±0.002^b,A^	0.006±0.001^b,AB^
		G	0.017±0.004^b,A^	0.012±0^ab,A^	0.012±0.008^b,A^	0.021±0^a,A^
		I	0.073±0.001^a,A^	0.017±0^ab,C^	0.033±0.013^a,B^	0.034±0.004^a,B^
Proline	M	U	0.165±0^a,A^	0.067±0.003^a,B^	0.011±0.011^c,C^	0.07±0.001^a,B^
		G	0.023±0.014^c,B^	0.01±0.001^b,B^	0.090±0.018^b,A^	0.002±0.001^b,B^
		I	0.130±0.003^a,B^	0.092±0.002^a,C^	0.168±0.035^a,A^	0.084±0.008^a,C^
	B	U	0.082±0.008^b,A^	0.062±0.019^a,A^	0.017±0.009^c,B^	0.063±0.006^a,A^
		G	0.109±0.006^ab,A^	0.007±0.006^b,B^	0.140±0.006^a,A^	0.008±0.005^b,B^
		I	0.128±0.004^a,B^	0.092±0^a,C^	0.170±0.006^a,A^	0.088±0.003^a,C^
**Essential amino acids (g/100 g dw)**						
Histidine	M	U	0.023±0.001^b,B^	0.005±0^b,C^	0.052±0.014^bc,A^	0.012±0.001b^c,BC^
		G	0.019±0^b,B^	0.010±0.001^b,B^	0.065±0.001^b,A^	0.019±0.001^b,B^
		I	0.070±0.001^a,B^	0.072±0.002^a,AB^	0.050±0.011^bc,C^	0.081±0.006^a,A^
	B	U	0.019±0.002^b,B^	0.005±0^b,C^	0.048±0.010^c,A^	0.003±0^c,C^
		G	0.022±0.001^b,B^	0.011±0^b,B^	0.090±0.001^a,A^	0.022±0^b,B^
		I	0.065±0.001^a,B^	0.067±0.001^a,B^	0.050±0.005^bc,C^	0.087±0.008^a,A^
Threonine	M	U	0.029±0^b,B^	0.008±0^b,B^	0.136±0.028^b,A^	0.014±0.002^b,B^
		G	0.032±0.003^ab,B^	0.004±0^b,C^	0.119±0.004^bc,A^	0.012±0.001^b,BC^
		I	0.057±0^ab,B^	0.042±0^a,B^	0.078±0.018^d,A^	0.049±0.006^ab,B^
	B	U	0.026±0.001^b,B^	0.007±0.002^b,B^	0.107±0.015^c,A^	0.024±0^b,B^
		G	0.030±0.001^b,B^	0.004±0.001^b,C^	0.196±0.011^a,A^	0.017±0^b,BC^
		I	0.056±0.001^a,A^	0.042±0^a,A^	0.054±0.008^d,A^	0.054±0.008^a,A^
Valine	M	U	0.052±0.001^b,B^	0.015±0^b,B^	0.343±0.049^a,A^	0.030±0.001^b,B^
		G	0.076±0.005^b,B^	0.011±0.001^b,C^	0.252±0.002^b,A^	0.033±0.001^b,BC^
		I	0.161±0.001^a,B^	0.125±0.006^a,B^	0.235±0.040^b,A^	0.153±0.019^a,B^
	B	U	0.049±0.003^b,B^	0.014±0^b,B^	0.249±0.026^b,A^	0.038±0.002^b,B^
		G	0.068±0.001^b,B^	0.012±0^b,C^	0.382±0.021^a,A^	0.037±0.001^b,BC^
		I	0.164±0^a,A^	0.126±0.001^a,A^	0.167±0.019^c,A^	0.160±0.022^a,A^
Methionine	M	U	0.008±0^b,B^	0.005±0^b,B^	0.091±0.008^a,A^	0.004±0.001^b,B^
		G	0.033±0.001^ab,B^	0.012±0.001^b,B^	0.097±0.001^a,A^	0.016±0.001^b,B^
		I	0.125±0.001^a,A^	0.123±0.005^a,A^	0.099±0.021^a,B^	0.124±0.020^a,A^
	B	U	0.010±0.001^b,B^	0.003±0^b,B^	0.101±0.007^a,A^	0.011±0^b,B^
		G	0.032±0^b,B^	0.010±0^b,B^	0.101±0.005^a,A^	0.017±0^b,B^
		I	0.115±0.003^a,A^	0.125±0.001^a,A^	0.113±0.016^a,A^	0.129±0.021^a,A^
Tryptophan	M	U	0.031±0.001^b,B^	0.012±0^b,B^	0.120±0.016^b,A^	0.017±0^b,B^
		G	0.031±0.005^b,AB^	0.012±0.004^b,B^	0.053±0^a,A^	0.019±0.001^b,B^
		I	0.099±0.001^a,A^	0.085±0.008^a,A^	0.111±0.023^a,A^	0.112±0.012^a,A^
	B	U	0.02±0.001^b,B^	0.009±0.001^b,B^	0.07±0.006^b,A^	0.015±0^b,B^
		G	0.021±0.001^b,B^	0.013±0.001^b,B^	0.067±0.005^a,A^	0.014±0.002^b,B^
		I	0.097±0.001^a,AB^	0.089±0^a,B^	0.099±0.014^a,AB^	0.119±0.017^a,A^
Phenylalanine	M	U	0.051±0.001^b,B^	0.026±0^b,B^	0.276±0.044^b,A^	0.038±0^b,B^
		G	0.127±0^b,B^	0.061±0.001^b,B^	0.275±0.004^b,A^	0.080±0.002^b,B^
		I	0.325±0.004^a,B^	0.388±0.006^a,A^	0.285±0.059^b,A^	0.368±0.042^a,A^
	B	U	0.050±0.004^b,B^	0.019±0^b,B^	0.246±0.028^b,A^	0.044±0.001^b,B^
		G	0.121±0.001^b,B^	0.061±0.002^b,B^	0.334±0.016^ab,A^	0.081±0.002^b,B^
		I	0.321±0.005^a,A^	0.424±0.002^a,A^	0.379±0.062^a,A^	0.400±0.066^a,A^
Isoleucine	M	U	0.027±0.001^b,B^	0.006±0^b,B^	0.194±0.034^ab,A^	0.015±0^b,B^
		G	0.040±0.002^b,B^	0.003±0^b,C^	0.147±0.002^b,A^	0.013±0.001^b,BC^
		I	0.120±0.001^a,A^	0.125±0.001^a,A^	0.120±0.028^b,A^	0.141±0.016^a,A^
	B	U	0.025±0.001^b,B^	0.006±0.001^b,B^	0.148±0.021^b,A^	0.019±0^b,B^
		G	0.032±0.001^b,B^	0.003±0.001^b,B^	0.221±0.01^a,A^	0.016±0.001^b,B^
		I	0.110±0.003^a,B^	0.125±0.001^a,AB^	0.111±0.020^b,B^	0.151±0.025^a,A^
Leucine	M	U	0.050±0.001^b,B^	0.015±0^b,B^	0.37±0.038^b,A^	0.028±0.001^b,B^
		G	0.119±0.004^b,B^	0.026±0.002^b,B^	0.326±0.001^b,A^	0.044±0.011^b,B^
		I	0.490±0.006^a,A^	0.502±0.025^a,A^	0.454±0.105^ab,A^	0.489±0.054^a,A^
	B	U	0.048±0.004^b,B^	0.014±0^b,B^	0.320±0.023^b,A^	0.040±0.001^b,B^
		G	0.118±0.001^b,B^	0.022±0.004^b,B^	0.414±0.021^ab,A^	0.059±0.018^b,B^
		I	0.496±0.006^a,A^	0.493±0.004^a,A^	0.485±0.081^a,A^	0.518±0.078^a,A^
Lysine	M	U	0.048±0.002^b,B^	0.013±0.001^b,B^	0.243±0.020^b,A^	0.028±0^b,B^
		G	0.059±0.002^b,B^	0.015±0.001^b,B^	0.226±0.001^b,A^	0.034±0^b,B^
		I	0.409±0.012^a,A^	0.287±0.013^a,A^	0.376±0.105^a,A^	0.312±0.045^a,A^
	B	U	0.043±0.009^b,B^	0.015±0.001^b,B^	0.236±0.039^b,A^	0.041±0.003^b,B^
		G	0.059±0.004^b,B^	0.014±0^b,B^	0.273±0.013^b,A^	0.046±0.005^b,B^
		I	0.397±0.013^a,A^	0.309±0.004^a,B^	0.339±0.070^ab,AB^	0.367±0.070^a,AB^

Mean ± standard deviation of three replicates. Different lowercase letter within the column indicates statistical differences among different digestion phases of the same chia fractions in each amino acid (*p* < 0.05, Tukey test). Different uppercase letter within the same row indicates statistical differences among chia samples at the same digestion phases in each amino acid (*p* < 0.05, Tukey test). Abbreviations: DDF, degummed-defatted flour; PC, protein concentrates; Alb, albumin; Glo, globulin; M, Mexican chia sample; B, British chia sample; U, undigested samples; G, endpoint of gastric digestion; I, endpoint of intestinal digestion.

Among the non-essential amino acids (NEAA), arginine and tyrosine emerged as the most abundant FAA in chia DDF, PC, Alb, and Glo. Regarding the influence of chia seeds origin on the free arginine content of chia ingredients, it was observed that Mexican chia samples showed higher arginine contents in comparison to their British counterparts before SGID. Conversely, the results exhibited the opposite trend after SGID. Among them, PC presented a substantial increase in arginine content in comparison to the undigested samples (19-fold for MPC and 21-fold for BPC), while MAlb and BAlb exhibited a relatively minor rise in arginine content after SGID, with fold changes of 1.4 and 1.8, respectively. Arginine is known for its potential role in preventing heart disease (Pszczola, 2000). Therefore, chia, being rich in arginine, could be considered as a beneficial dietary ingredient with potential to contribute to cardiovascular health. Likewise, a similar trend was observed for tyrosine with its content also showing an increase after digestion. Specifically, MPC and BPC demonstrated considerable changes, indicating 13-fold and 14-fold increases, respectively, compared to their respective undigested equivalents. In contrast, the Alb fraction, MAlb and BAlb, displayed more modest variations (1.4-fold and 1.7-fold increases, respectively). Relevant to this observation, Kowalska et al. (2022) conducted a study on chickpea and soy flours and reported significant levels of free tyrosine, with values of 0.024 and 0.023 g/100 g dw, respectively. These values were found to be lower than the results obtained in this study for the undigested MDDF and BDDF, with respective values of 0.042 and 0.04 g/100 g dw. The amino acids that appear to exert a limiting influence on protein content are NEAA, especially free cysteine and proline. Concentrations for these amino acids were the smallest before and after SGID. Cysteine, as one of the sulfur-containing amino acids, plays a significant role in preserving the tertiary and quaternary structure of proteins (Paredes-Lopez, 1991). On the other hand, proline functions as a signaling molecule, capable of modulating mitochondrial functions, influencing cell proliferation, cell death, and triggering specific gene expression.

Regarding essential amino acids (EAA), leucine is considered as the most abundant amino acid in chia samples before and after SGID, followed by lysine and phenylalanine. The leucine content varied among samples before digestion, ranging from 0.014 to 0.37 g/100 g dw, but reached similar values (0.454–0.517 g/100 g dw) after SGID in all samples. Particularly, MPC and BPC showed significant increases of 34-fold and 35-fold, respectively, after SGID, while DDF and Glo from both locations displayed over 10-fold increases. Alb showed minor increases of 1.2-fold and 1.5-fold for the Mexican and British samples, respectively. A similar trend was observed for phenylalanine and lysine from both Mexican and British samples after SGID, with PC, Glo, DDF, and Alb displaying substantial increases and reaching relatively consistent values ranging from 0.265 to 0.424 g/100 g dw for phenylalanine and 0.454–0.518 g/100 g dw for lysine. Remarkably, digested Mexican and British Alb exhibited the highest content for most of the EAA. In comparison to previous studies, the undigested DDF in this study showed higher levels of free phenylalanine (0.051 g/100 g dw for MDDF and 0.050 g/100 g dw for BDDF) than peanut (0.011 g/100 g dw) and pumpkin flours (0.009 g/100 g dw) (Kowalska et al., 2022). Furthermore, attention should be given to tryptophan, a precursor of serotonin, as its synthesis in the brain depends on the availability of dietary precursors (Friedman, 2018; Jenkins et al., 2016). Chia seeds displayed significant differences in individual FAA content compared to traditional wheat and cereals, as lysine and tryptophan are commonly limiting EAAs in corn, oats, and rice (Sytar et al., 2018; Mustafa et al., 2007; Sánchez-Velázquez et al., 2021). However, in this study, it was observed that chia proteins presented the lowest content in histidine and threonine before and after SGID. Histidine plays a crucial role in various physiological processes, including enzyme catalysis and metal ion coordination (Schneider, 1978; Fullenkamp et al., 2013), while threonine is essential for protein synthesis and contributes to immune function and intestinal health (Feng et al., 2013; Abbasi et al., 2014).

### 3.5 Changes in the protein profile of Mexican and British chia ingredients, Alb and Glo fractions during gastrointestinal transit

The changes in the protein profile of Mexican and British DDF, PC, Alb and Glo fractions during *in vitro* digestion was analyzed using SDS-PAGE as summarized in Fig. 1. Prior to digestion, both MDDF and BDDF showed similar band distributions. Two prominent bands were evident in MDDF and BDDF (Fig. 1A), with MW of 20 and 30 kDa, indicating the presence of albumin and/or glutelin (Orona-Tamayo et al., 2015; López et al., 2018b; Malik and Riar, 2022). More intense bands were observed in the range of 50–60 kDa, attributed to the presence of 11 S globulin. Under non-reducing conditions, the 11 S globulin can be resolved into monomers with MW from 50 to 60 kDa (Sandoval-Oliveros and Paredes-López, 2013; Kačmárová et al., 2016). It has been confirmed that 11 S globulin is the most abundant protein in chia seeds (López et al., 2018a; Wang et al., 2023). Additionally, DDF profiles exhibited bands of low MW (less than 20 kDa), likely originating from both Alb and the prolamin fraction (Orona-Tamayo et al., 2015). Furthermore, the presence of trypsin inhibitors also could contribute to the appearance of low MW bands (approximately 10, 15 and 22 kDa) (Jiménez-Munoz et al., 2022). Recently, the existence of ShTl (trypsin inhibitor) in chia seeds has been confirmed, presenting a MW of ∼11 kDa under non-reducing conditions. ShTl exhibited resistance to high temperature (100 °C) and acid-alkaline (pH 2–10) conditions (de Souza et al., 2022). Thus, enabling its protein extraction during sample preparation steps used in this study. Similarly, undigested PC (Fig. 1B) exhibited a similar band distribution profile as DDF, indicating PC encloses all the prominent proteins present in DDF.

According to previous studies (Sandoval-Oliveros and Paredes-López, 2013; Orona-Tamayo et al., 2015), undigested Alb does not show high intensity bands, particularly in the MW range between 60 and 220 kDa. Therefore, a tricine gel was employed for a higher electrophoretic resolution of low MW proteins present in Alb fraction. Differences in the protein profile between MAlb and BAlb were observed (Fig. 1C), similar to the findings reported by Grancieri et al. (2019c). In particular, the protein band of 20 kDa showed higher intensity in BAlb compared to MAlb. Additionally, MAlb displayed four intense bands at 3.5, 7, 10, and 13 kDa, while BAlb presented three intense bands at 3.5, 5, and 11 kDa. The variations in Alb protein profile related to chia seed origin could be attributed to genetic and environmental factors (Malik and Riar, 2022). The 13 kDa band observed in MAlb is similar in size to the ‘high cysteine’ 2 S albumin found in many oilseeds (Youle and Huang, 1981; Srdić et al., 2020). It is probable that 2 S albumins with MW between 7 and 9 kDa also exist in MAlb (Shewry and Pandya, 1999). Regarding Glo fraction, protein profile (Fig. 1D) closely resembled that of DDF and PC, indicating that Glo is the most abundant protein fraction in the chia samples.

All chia samples showed an extensive protein hydrolysis after the *in vitro* gastric digestion (Fig. 1). In fact, most high MW polypeptides were not visible at the end of the gastric stage in DDF, while low MW bands ≤20 kDa were persistent in the gastric and intestinal digestates regardless of chia seed origin (Fig. 1A). For PC, it was possible to conclude that under *in vitro* gastric conditions, only low MW polypeptides (≤10 kDa) persisted in the gastric digesta whereas polypeptides ranging from 3.5 to 60 kDa persisted at the end of intestinal stage (Fig. 1B). This observation may be attributed to the neutral pH during intestinal stage, which potentially facilitates the solubility of higher MW proteins, while at gastric stage the pH is acidic, thus promoting protein aggregation. Comparison of the bands under intestinal conditions between DDF and PC clearly demonstrate a difference in digestibility between both ingredients while no differences were observed related to chia seed origin. Alb fraction (Fig. 1C) showed persistent bands at 80 kDa and below 10 kDa in both Mexican and British samples after the gastric phase whose intensity was notably reduced at the end of the intestinal stage. For Glo fraction (Fig. 1D), two intense bands between 3.5 and 15 kDa persisted in the gastric digesta, while digestion resistant polypeptides with MW ranging from 5 to 30 kDa, with higher intensity in MGlo than BGlo were observed at the end of intestinal stage. Although the SDS-PAGE profile of chia proteins has been previously investigated, this study performs for the first time a characterization of protein digestion products from chia ingredients and protein fractions, thus, making comparisons with other studies not feasible.

### 3.6 Changes in the size distribution of peptides in Mexican and British chia ingredients, Alb and Glo fractions during gastrointestinal transit

**Figure 2:**
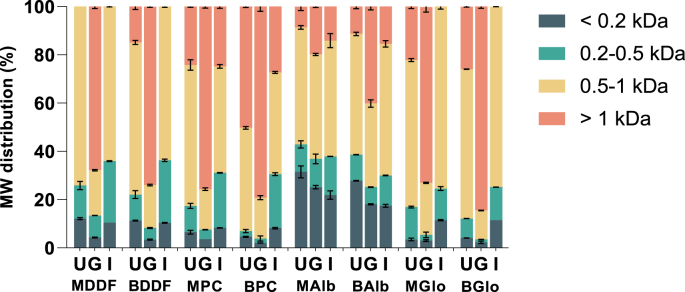
Peptide distribution (as ratio of total area eluted peak (%)) of soluble fractions of chia (*Salvia hispanica* L.) ingredients and protein fractions before and after SGID. Abbreviations: MDDF, Mexican degummed-defatted flour; BDDF, British degummed-defatted flour; MPC, Mexican protein concentrates; BPC, British protein concentrates; MAlb, Mexican chia albumin; BAlb, British chia albumin; MGlo, Mexican chia globulin; BGlo, British chia globulin; U, undigested sample; G, gastric digestion endpoint; I, intestinal digestion endpoint.

To better understand possible differences in the peptide distributions of the undigested samples, as well as the gastric and intestinal digestates, supernatants from the Mexican and British chia ingredients and protein fractions were analyzed by size exclusion chromatography. Representative elution profiles are shown in Fig. 2S. Under experimental conditions, the peptides present in the chia samples eluted in a MW range from 0.2 to 1 kDa. Four regions were quantified as percentage over total elution area (Fig. 2), reporting an elution of peptides in four different groups: >1 kDa (between 9.1 and 9.9 min), 0.5–1 kDa (10.0–13.6 min), 0.2–0.5 kDa (13.7–15.9 min), and <0.2 kDa (over 16 min). The distribution percentages of the different peptide sizes in chia samples are shown in Table 1S (Supplementary Material) and visualized in Fig. 2. Except for BPC, the undigested chia samples showed a predominant population of soluble peptides between 0.5 and 1 kDa, constituting 48–75 % of the total peptide fraction. Conversely, BPC mainly consisted of peptides exceeding 1 kDa, accounting for 50 % of total peptide fraction. While MAlb and BAlb presented a significantly high proportion of smaller peptides (<0.5 kDa) in comparison to other undigested chia samples, comprising 43 % and 39 % (Table 1S, *p* < 0.05), respectively. Additionally, a clear trend was observed, characterized by a decline in signals within the lower elution volume range (9–10 min) and a concurrent increase in signals within the higher elution volume range (10–16 min) relative to digestion time across most samples. This trend aligns well with the increasing presence of small peptides and decreasing amounts of high MW proteins and larger peptides.

At the end of gastric digestion, a discernible increase in the proportion of larger peptides (>1 kDa) and a reduction in the percentage of smaller peptide sizes (<0.2, 0.2–0.5, and 0.5–1 kDa) were observed (Fig. 2S and Fig. 2). Alb exhibited a distinctive attribute, characterized by its lower representation of larger peptides, constituting 21 % and 40 % for Mexican and British samples, respectively (*p* < 0.05, Table 1S). However, other samples exhibited analogous SEC profiles, defined by a reduced proportion of small peptide sizes, with samples from Mexico showing higher abundance of small peptides. This finding suggests a potentially elevated protein digestibility for Alb during gastric phase. After intestinal digestion, higher percentages of smaller peptides and a reduction in the proportion of peptides exceeding 1 kDa was observed across all samples. The intestinal digestates of PC and Alb from both locations exhibited a higher proportion of large peptides (25 % and 15.6 % for Mexican samples, 28 % and 16.2 % for British samples, respectively), while the digested DDF and Glo samples were characterized by a lack of larger peptides. These results imply an enhanced digestibility during the intestinal phase. Moreover, when considering the growing locations of both chia seeds, it becomes evident that the Mexican samples demonstrated a superior protein digestibility when compared to their British counterparts. This result differs from previous findings (Wang et al., 2023), in which British chia samples showed higher *in vitro* digestibility. This could be due to the different methods employed. Specifically, the INFOGEST, offers a digestive milieu more closely aligned with human metabolic process (Brodkorb et al., 2019), thus yielding a more precise and reliable outcome in the current study; while the *in vitro* protein digestibility method by Tinus et al. (2012), represents a more simple and rapid method to determine protein digestibility, this complicates the comparison of results since enzymatic conditions vary significantly across different methods.

### 3.7 Changes in antioxidant activity of Mexican and British chia ingredients, Alb and Glo fractions during gastrointestinal transit

**Figure 3:**
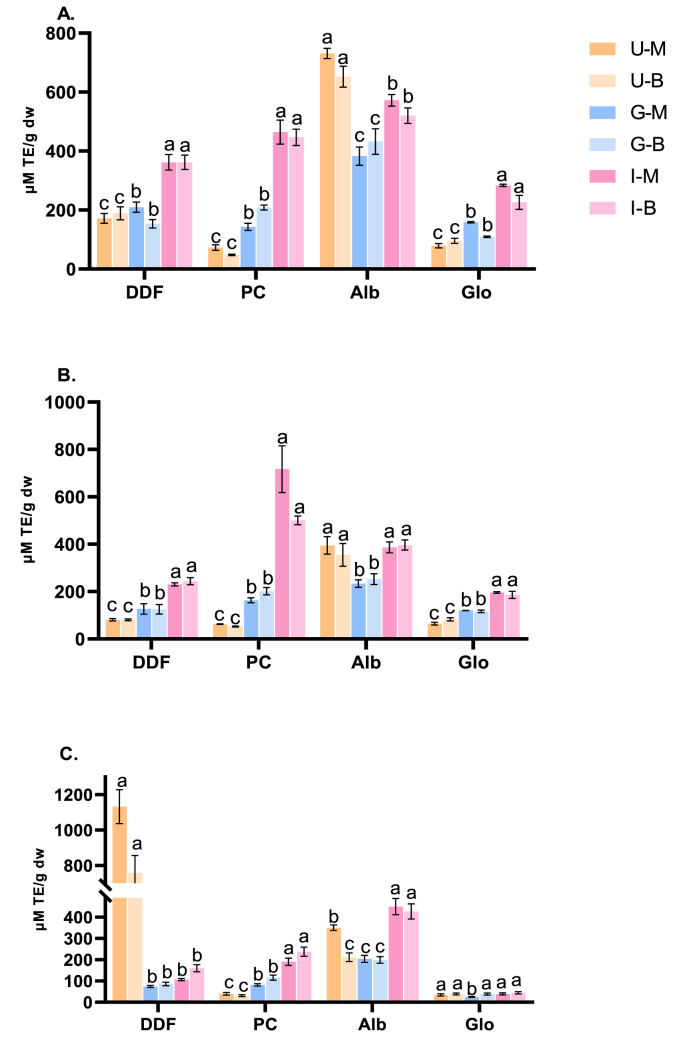
*In vitro* antioxidant activity measured in undigested chia (*Salvia hispanica* L.) ingredients and protein fractions and digestates after *in vitro* intestinal digestion. (A) Oxygen radical absorbance capacity (ORAC), (B) 2,2'-azino-bis (3-ethylbenzothiazoline-6-sulfonic acid) (ABTS) radical scavenging activity, and (C) 2,2-diphenyl-1-picrylhydrazyl (DPPH) radical scavenging activity. Abbreviations: DDF, degummed-defatted flour; PC, protein concentrates; Alb, chia albumin; Glo, chia globulin; M, Mexican chia samples; B, British chia samples; U, undigested; G, gastric phase; I, intestinal phase. Different lowercase letters within the groups indicates statistical differences among different digestion phases of the same chia sample (*p* < 0.05, Tukey test).

To elucidate the impact of chia processing, chia seed origin and gastrointestinal digestion on antioxidant activity, three *in vitro* assays were used: ORAC, ABTS and DPPH. ORAC measures the ability of a substance to scavenge free radicals and protect against oxidative damage (Kulczyński et al., 2019). As shown in Fig. 3A, prior to digestion, ORAC values of MDDF and BDDF were 172 and 189 μM TE/g dw, respectively. These values were lower compared to that reported for defatted Chilean chia seeds (517 μM TE/g) (da Silva Marineli et al., 2014). These differences in the antioxidant activity of chia seeds could be attributed to the influence of genetic and environmental factors as well as chia processing (Fernandes et al., 2023). In this regard, the removal of mucilage during processing could have reduced the antioxidant properties of chia flour (Fernandes et al., 2023). Among the samples, PC displayed the lowest ORAC values of 73 and 48 μM TE/g dw for Mexican and British samples, respectively. MAlb exhibited the highest ORAC activity of 731 μM TE/g dw, followed by BAlb (652 μM TE/g dw). While MGlo and BGlo showed 79 and 96 μM TE/g dw, respectively. The highest antioxidant activity observed for Alb was consistent with its higher phenolic content (TPC) (Wang et al., 2023). Phenolic compounds are potent free radical scavengers, effectively donating electrons to neutralize free radicals (Rizvi et al., 2010; Roy et al., 2010). Thus, ORAC values showed an increase in most samples as a function of digestion time. On the contrary, Alb fractions (MAlb and BAlb) showed a decrease of 48 % and 34 %, respectively, after gastric digestion. However, after intestinal digestion, MAlb exhibited a 49 % increase, and BAlb showed a 20 % increase. This phenomenon appears to be linked to the exposure of hydrophobic amino acids and the subsequent release of peptides resulting from intestinal proteolysis. Amino acids such as tryptophan exhibited a marked increase of 109 % and 48 % for MAlb and BAlb, respectively. Similarly, tyrosine showed increases of 24 % and 34 % for MAlb and BAlb, respectively. Moreover, phenylalanine presented an increase of 3.6 % and 13 % for MAlb and BAlb, while methionine displayed enhancements of 2.1 % and 12 % for MAlb and BAlb, respectively (Table 2) (Je et al., 2015; Sánchez-Velázquez et al., 2021). Despite the lower ORAC values in digested MAlb and BAlb compared to their undigested states, Alb still maintained the highest value compared to other chia samples (DDF, Alb, and Glo). In chia flours (DDF), ORAC values increased by 22 % in the MDDF but decreased by 19 % in BDDF after gastric digestion. After intestinal digestion, ORAC values of MDDF and BDDF increased by 101 % and 91 %, respectively, compared to the undigested samples. After digestion, PC exhibited a marked increase antioxidant activity, with a 537 % rise for MPC and 828 % rise for BPC. In a similar manner, digested MGlo displayed a 259 % increase in ORAC, and a 136 % increase for BGlo. For comparison purposes between the two seeds locations, it was observed that Mexican chia seeds exhibited higher ORAC values for the protein fractions (Alb and Glo), while both protein ingredients (DDF and PC) showed overall almost equal ORAC values.

ABTS assay is shown in Fig. 3B. Similar to ORAC assay, the highest ABTS value was observed in Alb samples before digestion, with values of 395 μM TE/g dw for MAlb and 355 μM TE/g dw for BAlb, respectively. While the lowest ABTS values were found in PC (69 and 54 μM TE/g dw for MPC and BPC, respectively). With the exception of Alb, ABTS activity increased in all samples during SGID. After gastric digestion, MAlb and BAlb exhibited a decrease of 41 % and 29 %, respectively, followed by an increase after intestinal digestion, reaching values of 396 and 397 μM TE/g dw, respectively. Alb, as previously mentioned, contains a significant amount of TPC before undergoing SGID. These TPC could donate electrons, neutralize free radicals, and halt further oxidative reactions (Schaich et al., 2015; Pérez-Jiménez and Saura-Calixto, 2008; Osman et al., 2006). The observed decline in antioxidant activity during the gastric phase can be attributed to diminished protein solubility under acidic pH conditions. The acidic conditions promotes protein dissociation, exposing binding sites for potential interactions with polyphenols through electrostatic affinities (Ozdal et al., 2013). Several globulin proteins, including albumins, form aggregates that interact with polyphenols, thereby reducing polyphenol solubility (Le Bourvellec and Renard, 2012; Quan et al., 2019), subsequently resulting in diminished antioxidant activity. On the contrary, the neutral pH in the intestinal phase enhances protein solubility, making proteins more accessible to digestive enzymes. Protein degradation in the intestinal phase facilitates the release of phenolics that were initially bound to proteins during the gastric phase, thus, contributing to the potential antioxidant properties of the metabolites formed in the digestive process (Stojadinovic et al., 2013; Quan et al., 2019). On the other hand, tryptophan, tyrosine, and sulfur amino acids (cysteine and methionine) are released during SGID and can exhibit enhanced antioxidant properties, as detected by the ABTS assay (Meucci and Mele, 1997). These amino acids’ antioxidant capacities contribute to the observed increase in ABTS values at the end of digestion.

In contrast to ORAC results, the highest ABTS values were found for MPC (760 μM TE/g dw) and BPC (508 μM TE/g dw) following the completion of digestion (Fig. 3B). Digested Alb and Glo demonstrated a significant increase of over 2-fold compared to their undigested counterparts (*p* < 0.05, Fig. 3B). This enhanced antioxidant ability of digested chia samples suggests the generation of new antioxidant peptides during SGID. In a study by Phongthai et al. (2018) on rice bran protein hydrolysates, it was found that peptides with lower MW (<3 kDa) exhibited higher ABTS activity. Similarly, this applies to all chia samples after SGID, where higher content of small peptides (0.2–1 kDa) was observed (section 3.6) with highest ABTS activity. These findings are in accordance to Foh et al. (2010), which demonstrated that protein hydrolysates from Tilapia (*Oreochromis niloticus*) with MW < 1 kDa exhibited higher efficiency in scavenging ABTS radicals. Similarly, Feng et al. (2018) confirmed the presence of two antioxidant peptides (VYTE and VSAFLA) from Chinese chestnut (*Castanea mollissima Blume*) protein hydrolysate with MW of 590.2 Da and 606.3 Da, respectively, which displayed the highest ABTS radical scavenging capacity.

The ability of chia samples to scavenge DPPH radicals was assessed, and the results are presented in Fig. 3C. Undigested MDDF and BDDF displayed the highest DPPH values before SGID (1132 and 759 μM TE/g dw, respectively, *p* < 0.05). This may be attributed to the presence of phenolics in DDF (Wołosiak et al., 2022). The previous study confirmed that MDDF and BDDF contains 629 and 580 mg gallic acid/100 g dw of phenolic compounds, respectively (Wang et al., 2023). Furthermore, phytic acid is recognized as a natural plant antioxidant that has the capacity to chelate metal ions like iron and copper, forming stable complexes (Graf and Eaton, 1990; Graf et al., 1987). In the prior study, MDDF and BDDF exhibited the highest phytic acid content among the chia samples, with values of 2.8 and 2.4 g/100 g dw, respectively (Wang et al., 2023). The abundance of phytic acid in these samples might have contributed to the high DPPH scavenging activity of DDF. In line with this, Khattab et al. (2010) reported that phytic acid demonstrated superior DPPH-scavenging activity compared to tannins in canola (*Brassica napus* L.) and Indian mustard (*Brassica juncea* L.). The DPPH radical scavenging ability of MPC and BPC (40 and 31 μM TE/g dw) was lower as compared to DDF, respectively. MGo and BGlo exhibited similar DPPH scavenging activity (39 and 36 μM TE/g dw, respectively) to PC, whereas Alb showed higher scavenging capacity than Glo and PC, with values of 350 and 212 μM TE/g dw for MAlb and BAlb, respectively (*p* < 0.05, Fig. 3C). After gastric digestion, the DPPH radical scavenging ability greatly decreased in DDF (93 % for MDDF and 89 % for BDDF). Once digestion was completed, MDDF and BDDF displayed increases of 43 % and 86 %, respectively. Similarly, MAlb and BAlb antioxidant activity decreased after gastric digestion (42 % and 6 %, respectively). Subsequently, after intestinal digestion, an evident increase of 121 % and 115 % was observed for MAlb and BAlb, respectively, indicating their enhanced DPPH radical scavenging ability. This finding aligns with the results reported by (Grancieri et al., 2019b). A similar trend was observed in Glo, with a decrease of 24 % and 9 % in Mexican and British samples, respectively, after gastric digestion, and subsequent increases of 40 % and 19 % after intestinal digestion were observed. Furthermore, the DPPH values of PC increased during SGID, with digested MPC and BPC showing 378 % and 668 % increases compared to undigested sample. This could be attributed to the high content of aromatic amino acids (phenylalanine, tryptophan, and tyrosine) present in PC (Wang et al., 2023). The cleavage of peptide bonds between hydrophobic and preferably aromatic amino acids during SGID, results in increased hydrophobicity, allowing PC released peptides to react effectively with DPPH radicals (Phongthai et al., 2018; Gulcin, 2020).

**Figure 4:**
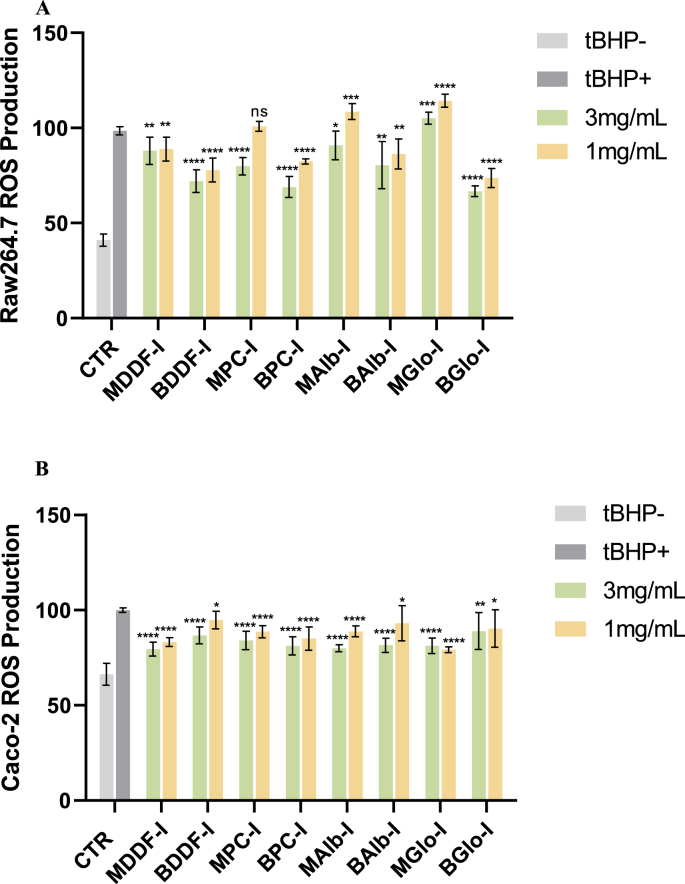
Cellular antioxidant activity measured in chia samples digestates after *in vitro* intestinal digestion. A) Production of intracellular reactive oxygen species (ROS) in *t*-BHP challenged RAW264.7 macrophages pretreated with intestinal digests. B) Production of intracellular ROS in t-BHP challenged Caco-2 cells pretreated with intestinal digests. Cells were induced into an oxidative stress (tBHP+) after the exposure at different concentrations of intestinal digests (1 and 3 mg/mL) for 20 h. Non-challenged cells (tBPH-) were used as negative control. Results are presented as mean ± SD (n = 8). *p* < 0.05 is considered significant (significance is denoted as follows: ns no significance; *p ≤ 0.05; **p ≤ 0.01; ***p ≤ 0.001; ****p ≤ 0.0001); *vs*. tBHP+ group. Abbreviations: MDDF, Mexican degummed-defatted flour; BDDF, British degummed-defatted flour; MPC, Mexican protein concentrates; BPC, British protein concentrates; MAlb, Mexican chia albumin; BAlb, British chia albumin; MGlo, Mexican chia globulin; BGlo, British chia globulin; I, intestinal phase; tBHP+, cells treated with t-BHP; tBHP-, untreated cells.

Two cellular models were also used to assess the antioxidant effect of intestinal digestates of chia ingredients and protein fractions. The effect of DDF, PC, Alb and Glo intestinal digestates on the modulation of intracellular ROS production in oxidative stressed RAW264.7 macrophages and Caco-2 intestinal cells is presented in Fig. 4. The addition of *t*-BHP triggered oxidative stress in macrophages (tBHP+), causing an increase in intracellular ROS production in comparison to the untreated cells (tBHP–) after 3 h of exposure (Fig. 4A). The results indicated that most of the tested intestinal digestates reduced dose-dependently oxidative stress in RAW264.7 cells, with the exception of MGlo. These results confirm that the protein digestion products maintain their antioxidant activity after gastrointestinal digestion. At the highest concentration tested (3 mg/mL), British intestinal digestates demonstrated a markedly superior ROS scavenging capability (*p* < 0.05, Fig. 4A) than the Mexican counterparts. BGlo showed the highest inhibition of intracellular ROS production (32 %), followed by BPC and BDDF (30 % and 27 %, respectively) whereas BAlb exerted the lowest reduction of ROS intracellular levels (18 %). A recent study from Villanueva-Lazo et al. (2022) showed that chia protein hydrolysates, derived from Mexican chia protein isolate after treatment with Alcalase exhibited ROS scavenging capability in human monocyte-macrophage plasticity response.

Regarding Caco-2 intestinal cells, the exposure to *t*-BHP for 3 h (tBHP+) increased ROS levels as compared to non-treated cells (tBHP−). All the digested Mexican and British (Fig. 4B) chia protein ingredients and protein fractions scavenged ROS, presenting similar ROS levels at varying concentrations (1 and 3 mg/mL). The findings suggest that both digested Mexican and British chia protein ingredients and protein fractions can effectively inhibit oxidative stress by reducing ROS production, especially at the highest tested concentrations (3 mg/mL). The observed antioxidant effects of the digested chia ingredients and protein fractions are likely attributable to the collective presence of phenolic compounds and specific peptides/amino acids. Previous studies highlighted the capacity of phenolic compounds to modulate cellular redox states and safeguard cells against oxidative damage by scavenging free radicals and chelating metal ions (Martemucci et al., 2022). These compounds also have the capacity to influence the activity of antioxidant enzymes and regulate signaling pathways involved in the cellular response to oxidative stress (Kučera et al., 2014). Amino acids such as cysteine serves as a precursor for glutathione, a potent intracellular antioxidant (Guru et al., 2021). Other amino acids, such as methionine, tryptophan, tyrosine, and proline are other amino acids known for their antioxidant capabilities (Levine et al., 2000; Atmaca, 2004; Brandelli et al., 2015). Moreover, certain peptides derived from chia proteins have been recognized as antioxidants. Madrazo and Campos (2022) demonstrated that the antioxidant activity of peptides, KLLKKYL, KKLLKI, YACLKVK, and KLKKNL, derived from a chia peptide fraction F < 1 kDa, obtained by *in silico* approaches, indicated that both optimized and chia-derived peptides contain amino acid residues associated with antioxidant activity, notably YACLKVK. These findings emphasize the beneficial influence of the bioactive compounds in chia seeds on managing oxidative stress and underscore their potential as therapeutic agents for diseases associated with oxidative damage.

### 3.8 Changes in the anti-inflammatory activity of Mexican and British chia ingredients, Alb and Glo fractions during gastrointestinal transit

**Figure 5:**
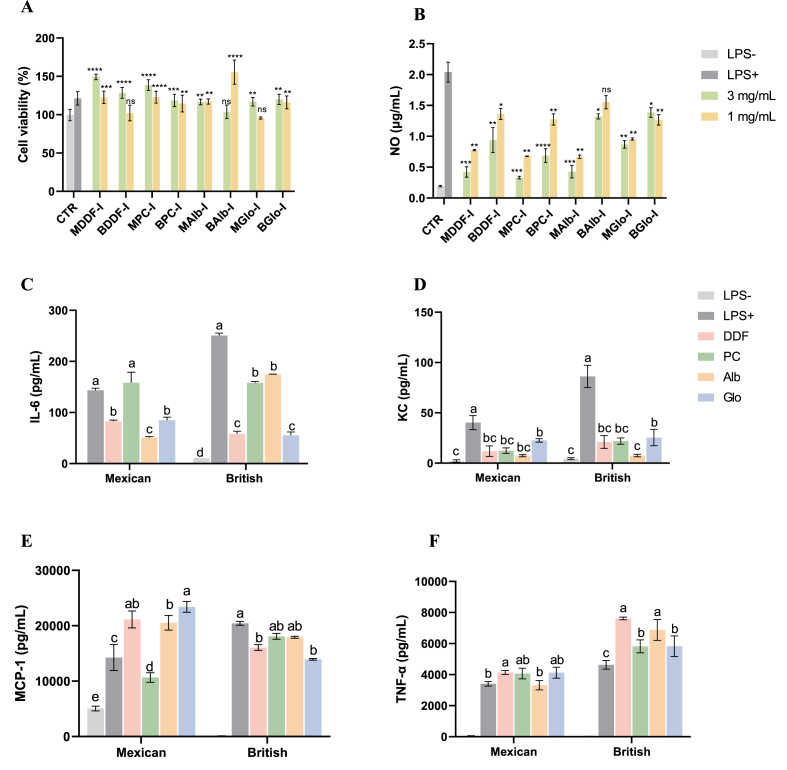
Anti-inflammatory activity measured in chia samples digestates after *in vitro* intestinal digestion. Effect of different concentrations (1 and 3 mg/mL) of intestinal digests from Mexican and British chia samples over the viability of RAW264.7 macrophage **(A)** Concentration of nitric oxide (B) and pro-inflammatory cytokines interleukin (IL)-6 (C), chemokine (C-X-C motif) ligand 1 (KC) (D) monocyte Chemoattractant Protein-1 (MCP-1) (E) Tumor Necrosis Factor-α (TNF-α) (F) released in the extracellular media by activated cells with LPS. Cells were induced into an inflammatory state (LPS+) in the presence of different concentrations of intestinal digests (1 and 3 mg/mL for NO and 3 mg/mL was selected to measure KC, IL-6, MCP-1, and TNF-α). Non-activated cells (LPS-) were used as negative control. Results are presented as mean ± SD (n = 5). *P* < 0.05 is considered significant (significance is denoted as follows: ns no significance; *p ≤ 0.05; **p ≤ 0.01; ***p ≤ 0.001; ****p ≤ 0.0001); (A) *vs*. LPS- group; (B) *vs*. LPS+ group (unpaired two-tailed *t* test). Different lowercase letters within the groups in (C, D, E, and F) indicates statistical differences among different digestion phases of the same chia sample (*p* < 0.05, Tukey test). Abbreviations: MDDF, Mexican degummed-defatted flour; BDDF, British degummed-defatted flour; MPC, Mexican protein concentrates; BPC, British protein concentrates; MAlb, Mexican chia albumin; BAlb, British chia albumin; MGlo, Mexican chia globulin; BGlo, British chia globulin; I, intestinal phase.

Macrophages are implicated in inflammatory responses, as these secrete an array of mediators such as cytokines, chemokines, and adhesion molecules (Grancieri et al., 2019c). Upon exposure to certain stimuli, such as lipopolysaccharides (LPS), macrophages activate though Toll-like receptor 4, the secretion of cytokines including IL-6, KC, Monocyte Chemoattractant Protein-1 (MCP-1), and Tumor Necrosis Factor-alpha (TNF-α) (Tucureanu et al., 2018; Araiza-Calahorra et al., 2022). To assess the putative anti-inflammatory capacity of Mexican and British chia digested proteins and fractions, these were assessed at different concentrations in LPS-challenged RAW264.7 macrophages. Fig. 5A shows cell viability after cell exposure to different doses of intestinal digestates. The results revealed that digested chia samples did not show cytotoxic effects.

Nitric oxide (NO) can activate tissue damage and DNA injury at sites of inflammation (Conforti and Menichini, 2011). In response to LPS exposure, most samples reduced NO release in RAW 264.7 cells in a dose-dependent manner. As shown in Fig. 5B, for Mexican digests, treatment with MPC (3 mg/mL) yielded the lowest NO production (0.33 μg/mL), thus showing a maximum NO inhibition of 84 %, followed by MDDF and MAlb (79.4 % and 78.9 %) (*p* < 0.05, Fig. 5B), respectively. Similar findings were observed for British chia digests; BPC exhibited the maximum NO inhibition of 66 % at 3 mg/mL, followed BDDF (54 %) and BAlb (35 %) (*p* < 0.05, Fig. 5B). However, the maximum NO inhibition for BGlo was observed at 1 mg/mL with a value of 38 %. Given that 3 mg/mL was the most effective concentration to reduce NO production, therefore, this concentration was employed to evaluate cytokine production (IL-6, KC, MCP-1, and TNF-α, Fig. 5C–F). IL-6 production was significantly reduced by both Mexican and British chia samples (*p* < 0.05, Fig. 5C), being MAlb and BGlo the samples exerting maximum inhibitions of 65 % and 78 %, respectively (Fig. 5C). Additionally, BDDF exhibited comparable IL-6 inhibition to BGlo, displaying a significant reduction of 74 % (*p* > 0.05, Fig. 5C). Both MAlb and BAlb yielded the lowest KC production (Fig. 5D), thus, showing the highest inhibitions of 82 % and 91 %, respectively. MCP-1 is recognized as a chemotactic cytokine, functioning as a pivotal driver of monocyte chemotaxis by recruiting additional monocytes to the site of inflammation (Bianconi et al., 2018). Simultaneously, TNF-α serves as a leading inflammatory mediator that macrophages secrete when stimulated by LPS (Takashiba et al., 1999; Grancieri et al., 2022). Moreover, a different behavior was observed in MCP-1 and TNF-α inhibition between Mexican and British chia samples (Fig. 5E and F, respectively). Among the Mexican samples, only PC showed inhibition of MCP-1 secretion (25 % inhibition, Fig. 5E), while all other samples did not contribute positively to the reduction of MCP-1 production. Similarly, MAlb was the only digestate capable to inhibit slightly TNF-α production (2.6 %) (Fig. 5F). Intestinal digests of British chia demonstrated a positive effect on the inhibition of MCP-1 production, with BGlo being the most potent, reducing this cytokine by 32 %. However, they did not effectively inhibit TNF-α production. Grancieri et al. (2022) reported different findings when examining the impact of digested Brazilian chia protein, which notably resulted in a reduction of TNF-α expression (data not shown).

The anti-inflammatory efficacy of plant-derived bioactive peptides is intricately influenced by their MW and amino acid sequence. Previous reviews have demonstrated that peptides with lower MW (<1 kDa) exhibit higher anti-inflammatory activity (Liu et al., 2022). Notably, investigations have identified plant-derived bioactive peptides with MW of approximately 0.5 kDa (∼5 amino acid residues) as possessing the most potent anti-inflammatory attributes (Craik et al., 2013). This observation aligns with the findings presented in section 3.6, where it was observed that digestion of MPC and BPC released peptides of smaller MW (<0.6 kDa), correlating with superior NO inhibition capacity. Similar conclusions were observed by Saisavoey et al. (2021), in which a bee pollen hydrolysate with a MW below 0.65 kDa showed the highest NO inhibitory activity.

In addition to MW, the anti-inflammatory properties of food-derived peptides are closely linked to their amino acid composition. Hydrophobic amino acids have been consistently identified as key contributors to the anti-inflammatory effects of peptides (Guha and Majumder, 2019). The mechanism underlying this effect involves the binding of hydrophobic amino acids to LPS molecules, resulting in the formation of peptide-LPS complexes that counteract LPS-induced inflammatory responses. Moreover, these hydrophobic amino acids are capable of scavenging LPS by inducing cell membrane charge reversal, further mitigating inflammation (Singh et al., 2017). Among these amino acids, leucine plays a pivotal role in enhancing the anti-inflammatory activity, with the presence of tryptophan and phenylalanine further augmenting this effect (Liu et al., 2022). Consequently, as elucidated in section 3.4, the high content of these hydrophobic amino acids in chia seeds is potentially responsible for their enhanced anti-inflammatory properties, distinguishing them from other traditional cereals and oilseeds.

Beyond hydrophobic amino acids, positively charged amino acids, such as lysine and arginine, have emerged as important contributors to the improved anti-inflammatory activity of plant-derived peptides **(**section 3.4). For instance, pure peptides, NSPGPHDVALDQ and RMVLPEYELLYE, isolated from Brazil chia seeds, have shown notable inhibitory effects on NO, PGE_2_, and TNF-α in RAW264.7 cells, with one of these peptides containing arginine in its amino terminal (Grancieri et al., 2021). Lysine has been found to be prevalent in most anti-inflammatory peptides derived from various plant sources. Oligopeptides KLRSRNLLHPT and TNGRHSAKKH, derived from bee pollen, have been demonstrated to inhibit the expression of COX-2, iNOS, IL-6, and TNF-α in RAW264.7 macrophages (Saisavoey et al., 2021). Likewise, green tea peptides (LAEQAER, VECTIPK, DAYVGDEAQSK, and MASLALK) have been shown to reduce iNOS and TNF-α in diabetic mice, with three of these peptides containing lysine (Chen et al., 2022).

Given the complexities governing the anti-inflammatory potential of plant-derived bioactive peptides, it is crucial to conduct further research to explore and identify such peptides in chia seeds, aiming for a comprehensive understanding of their anti-inflammatory mechanisms.

## 4 Conclusion

Our study undertook a thorough exploration of chia proteins' behavior during gastrointestinal transit, considering critical factors such as the geographic origin of chia seeds and the effects of various processing techniques. The investigation revealed distinctive digestion patterns for proteins derived from chia seeds sourced from different locations (UK and Mexico) and subjected to diverse extraction and fractionation methods. While similarities were observed in the breakdown of certain proteins in DDF, PC, and Glo, significant variations emerged in the bioaccessible content of protein, peptides and free amino acids as well as the size peptide distribution. These differences carry significant implications for the antioxidant and anti-inflammatory activities of the resultant intestinal digestates. Our study showcased the capability of digested chia protein ingredients and fractions to mitigate reactive oxygen species (ROS) production and reduce pro-inflammatory cytokine levels. Notably, British chia samples exhibited pronounced anti-inflammatory properties, emphasizing their promising role as healthier ingredients. In summary, the favorable protein content, digestibility, and potent antioxidant and anti-inflammatory attributes of chia, position this emergent seed as a compelling option for developing dietary strategies targeting oxidative damage and inflammation-related disorders. The insights gained from this investigation open avenues for further research, including the identification of specific bioactive peptides responsible for these health benefits and their innovative applications in functional foods and nutraceuticals.

## CRediT authorship contribution statement

**Yan Wang:** Validation, Formal analysis, Investigation, Writing – original draft, Visualization. **Alan Javier Hernández-Alvarez:** Conceptualization, Methodology, Validation, Investigation, Resources, Data curation, Writing – review & editing, Visualization, Supervision, Project administration, Funding acquisition. **Francisco M. Goycoolea:** Validation, Writing – review & editing. **Cristina Martínez-Villaluenga:** Conceptualization, Methodology, Validation, Investigation, Resources, Data curation, Writing – review & editing, Visualization, Supervision, Project administration, Funding acquisition.

## Declaration of competing interest

The authors declare that they have no known competing financial interests or personal relationships that could have appeared to influence the work reported in this paper.

## Data Availability

No data was used for the research described in the article.
