# Supplementary material for: A comparative study of the digestion behavior and functionality of protein from chia (Salvia hispanica L.) ingredients and protein fractions
Source: Curr Res Food Sci. 2024 Jan 24;8:100684. doi: 10.1016/j.crfs.2024.100684 (PMC10845256; doi:10.1016/j.crfs.2024.100684)
Supplement: Multimedia component 1 [file mmc1.docx]

**Figure 1S.** Comparison of total protein content in undigested Mexican and British chia (*Salvia hispanica* L.) ingredients and protein fractions. Abbreviations: DDF, degummed-defatted chia flour; PC, protein concentrate; Alb, albumin; Glo, globulin. Different lowercase letter within each sample group indicates statistical differences among the same chia samples from different locations (*p* < 0.05, Tukey test).


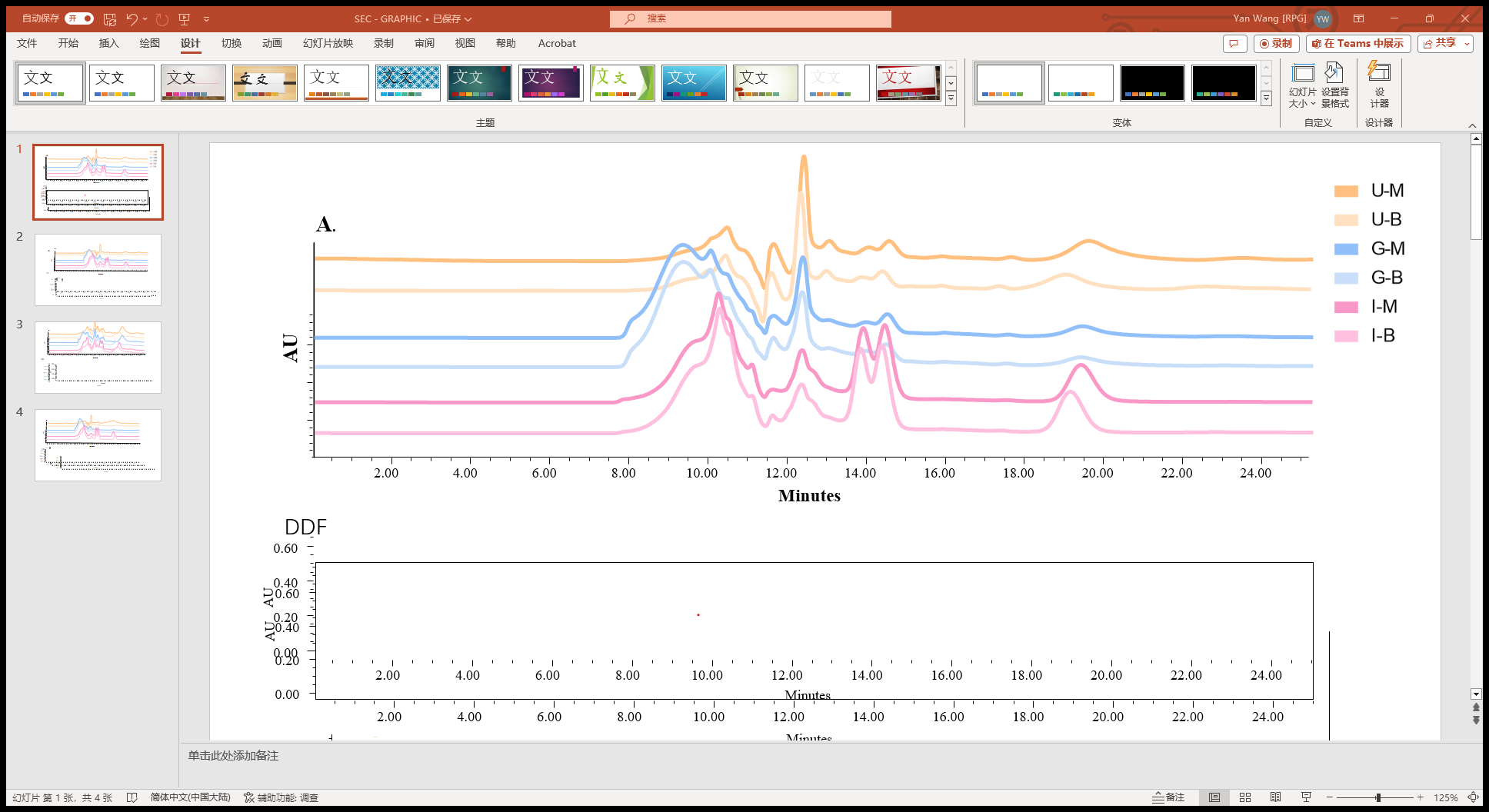


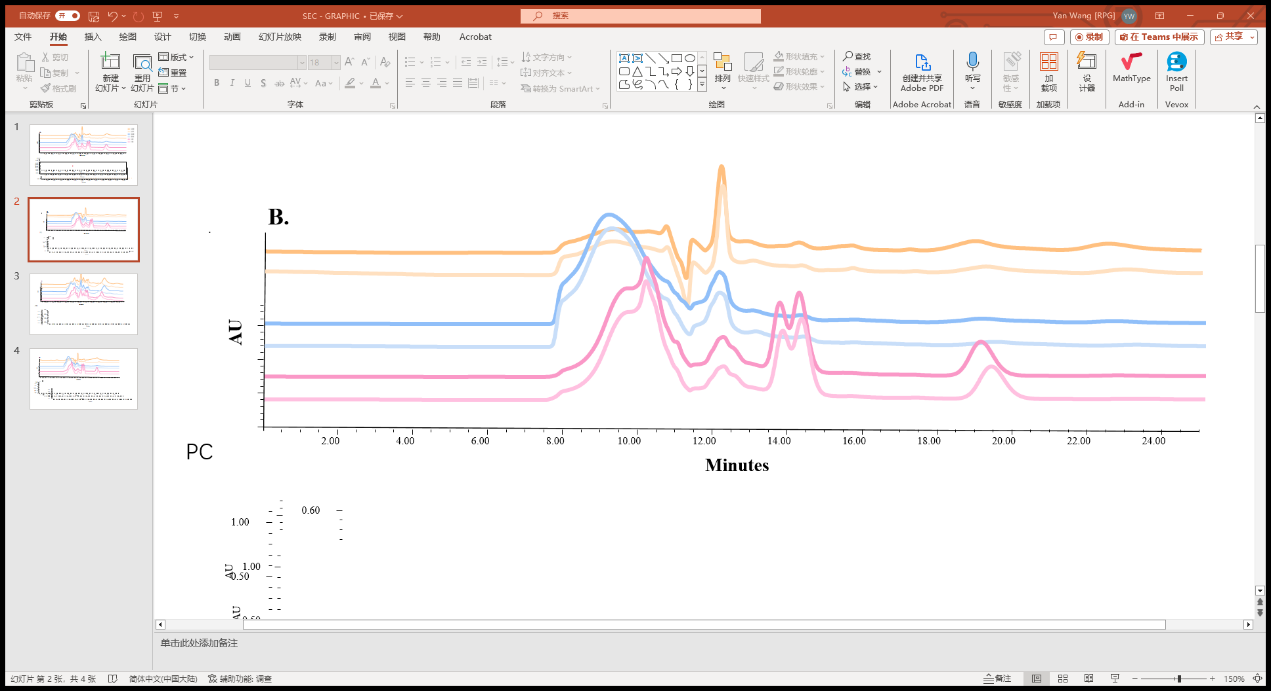


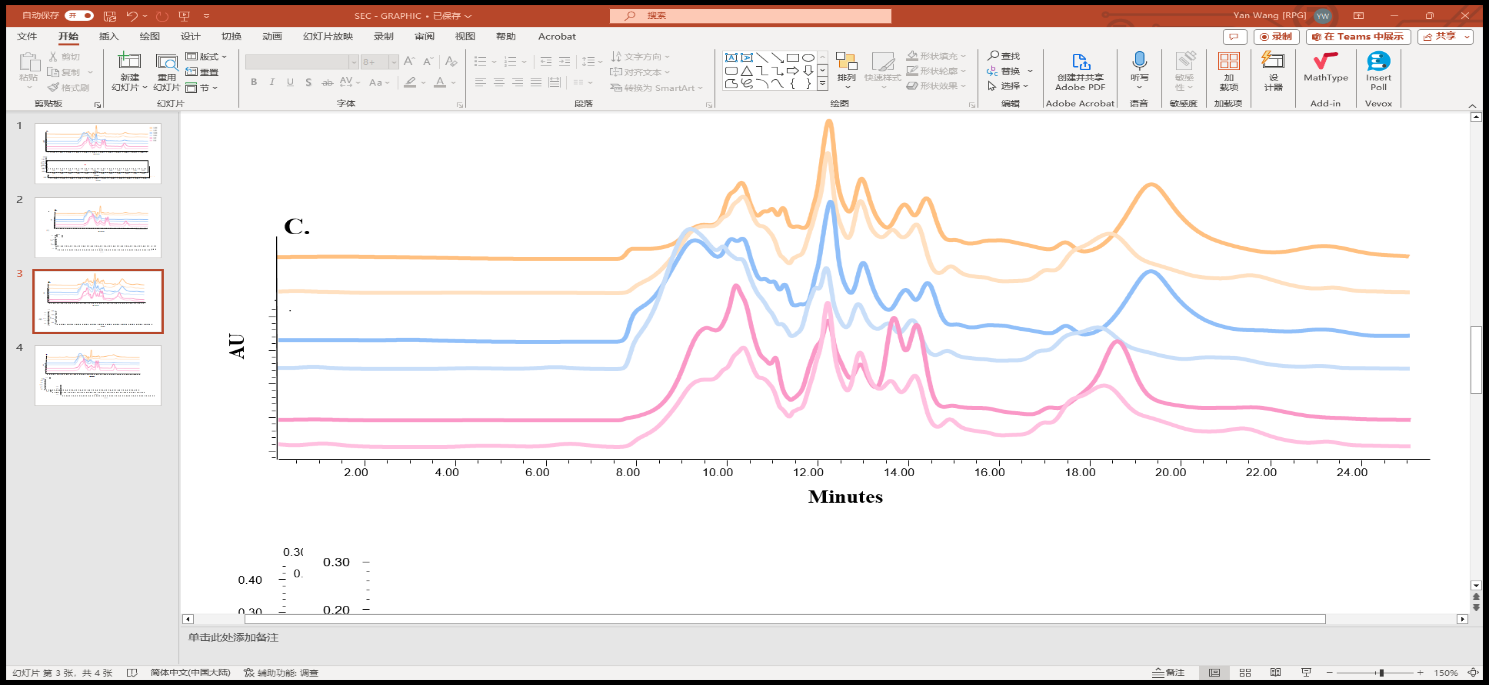


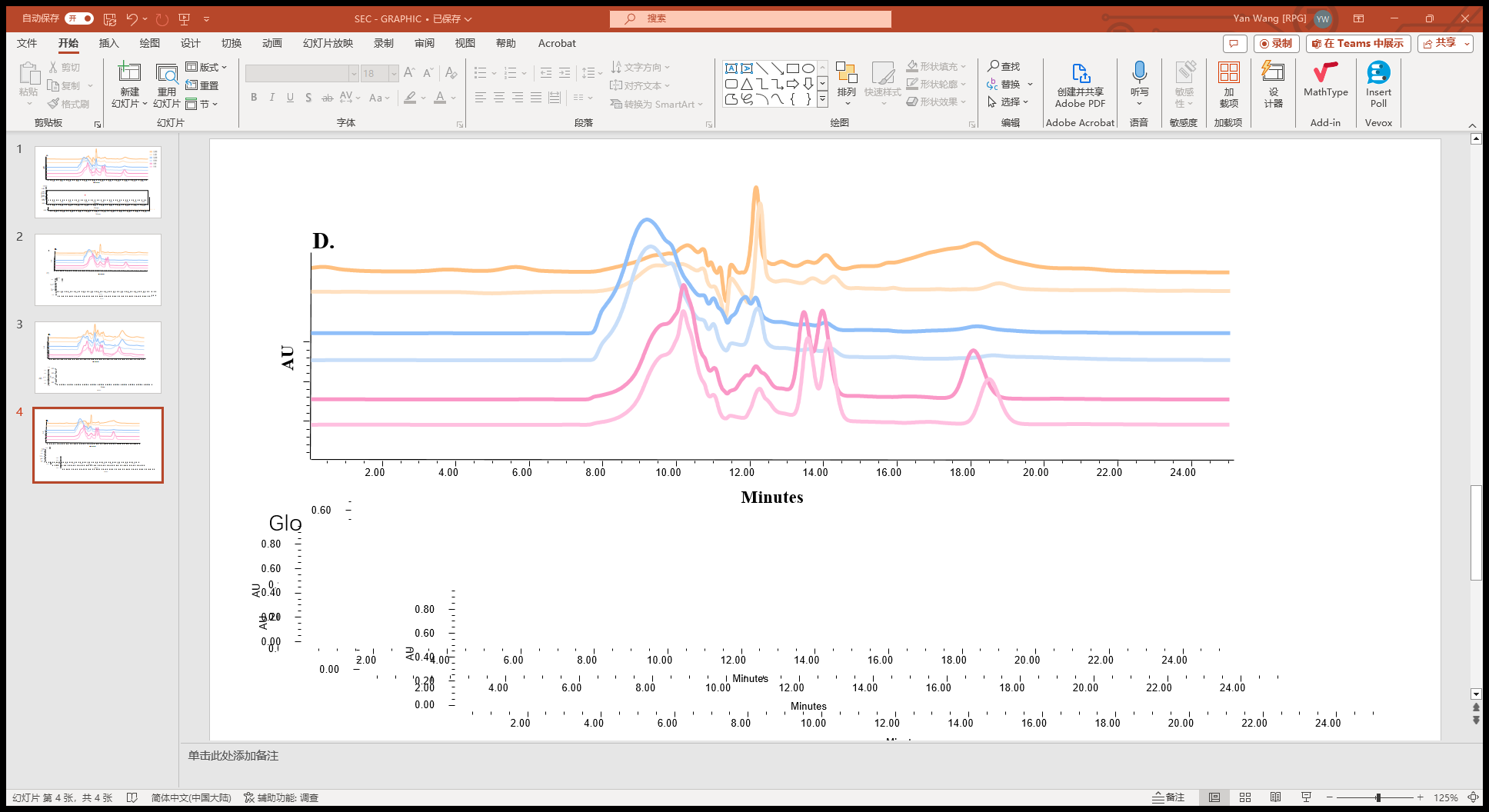


**Figure 2S**. SEC profile at 214 nm for undigested Mexican and British chia (*Salvia hispanica* L.) samples and their digestates after gastric and intestinal digestion. **A)** degummed-defatted flour; **B)** protein concentrate; **C)** albumin, **D)** globulin. Abbreviations: M, Mexican chia samples; B, British chia samples; U, undigested; G, gastric phase; I, intestinal phase.

A.


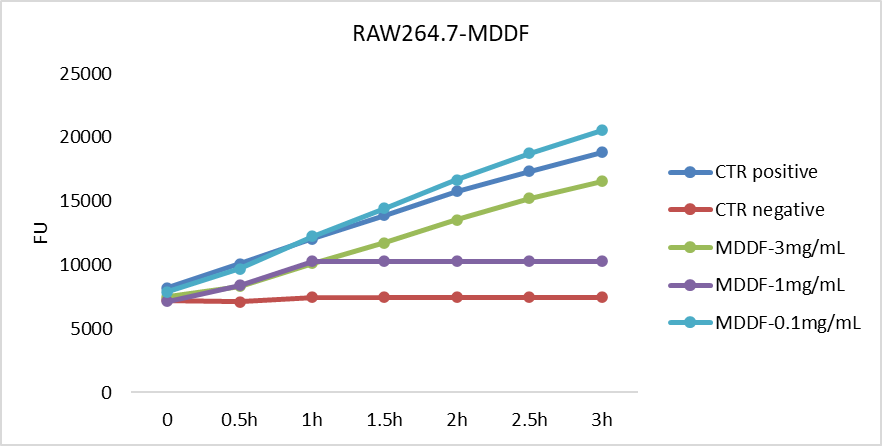

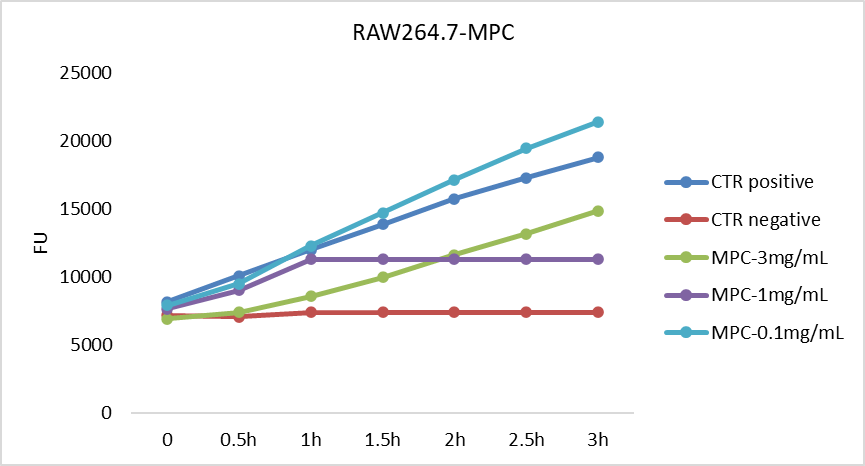


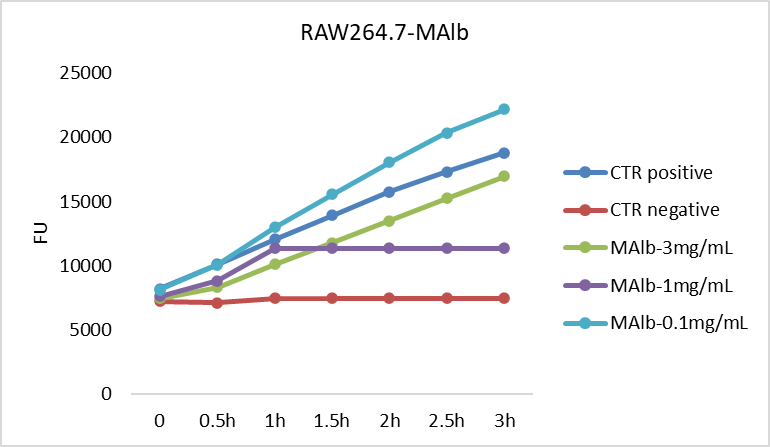

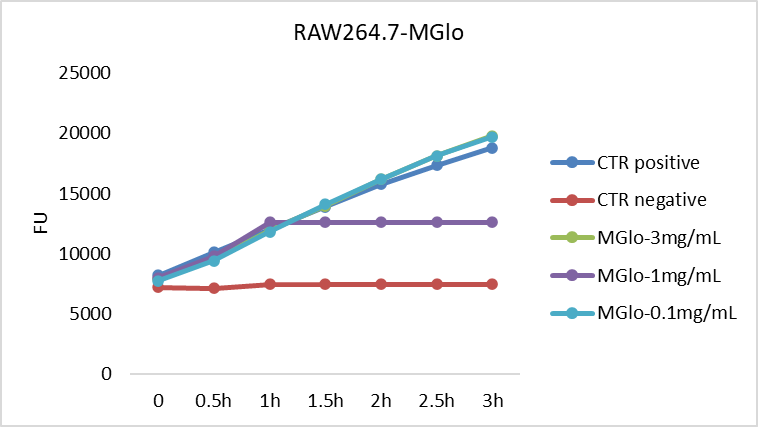


B.


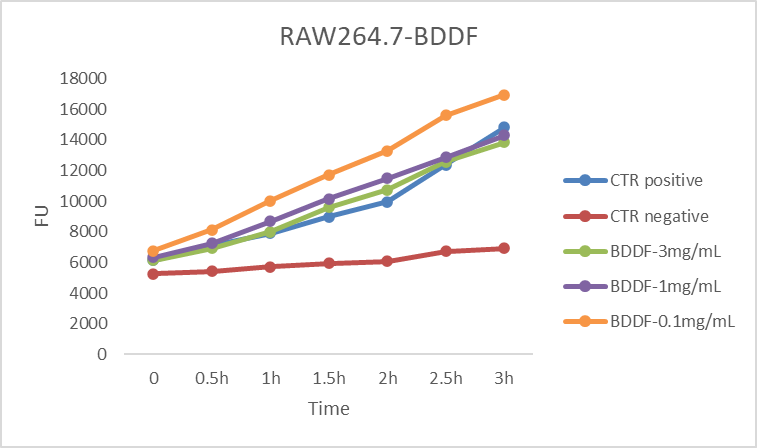

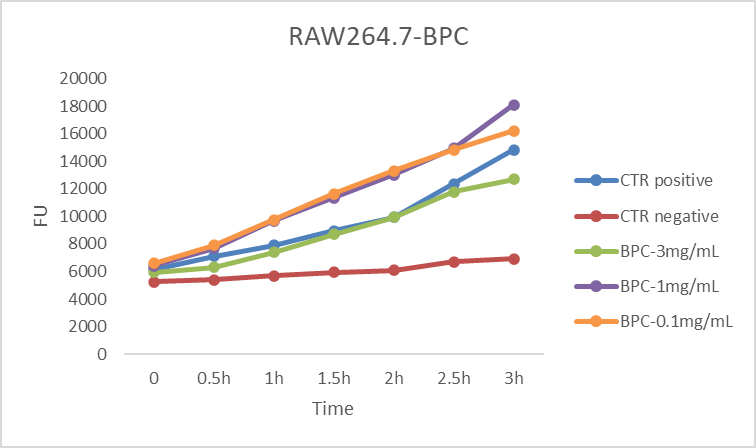


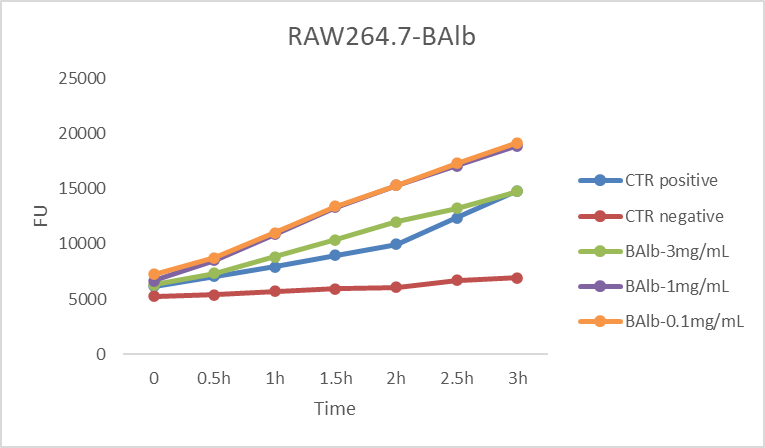

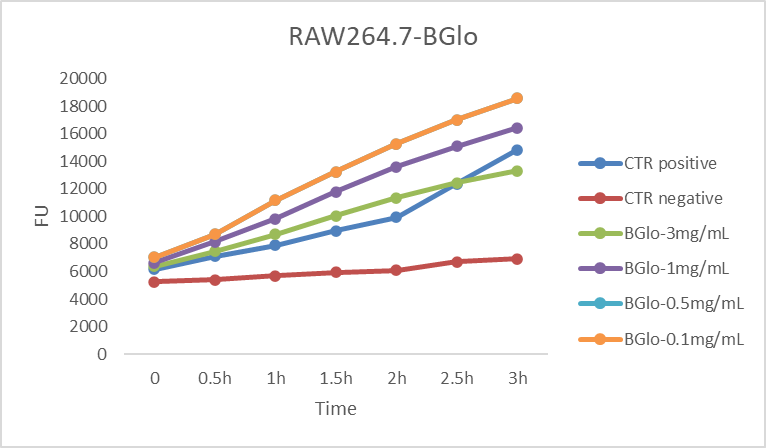


C.


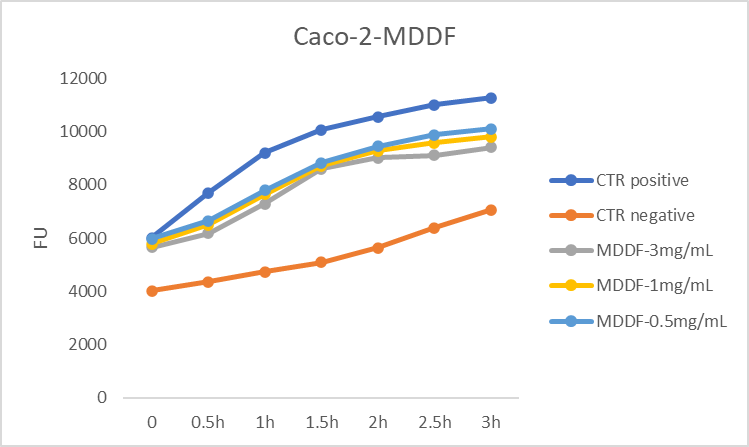

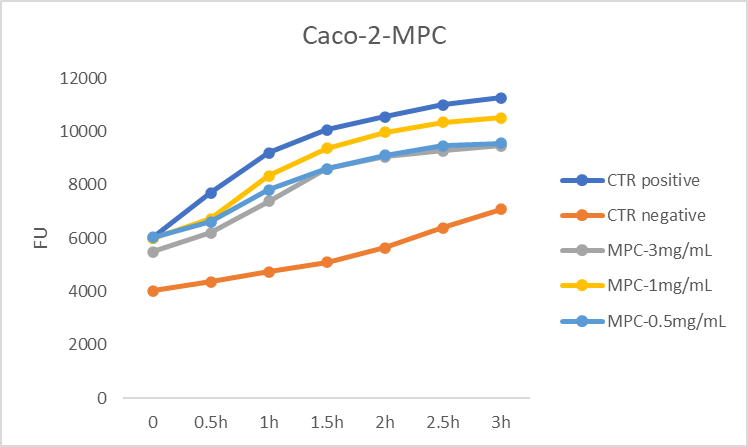


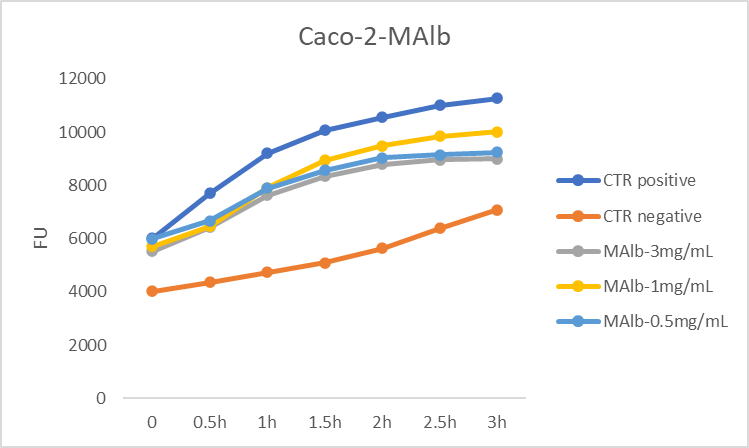

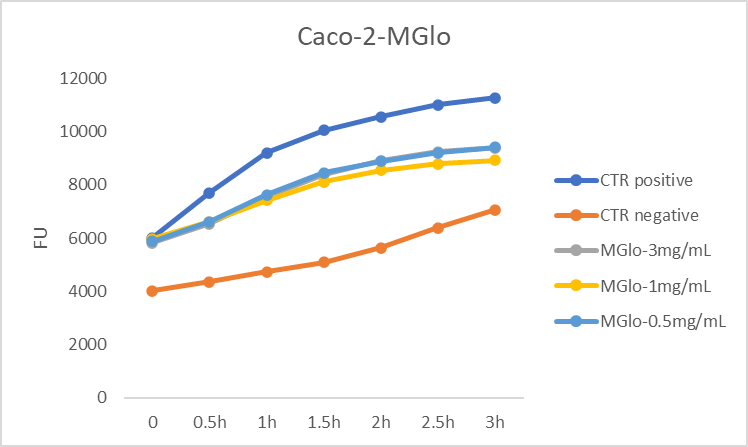


D.


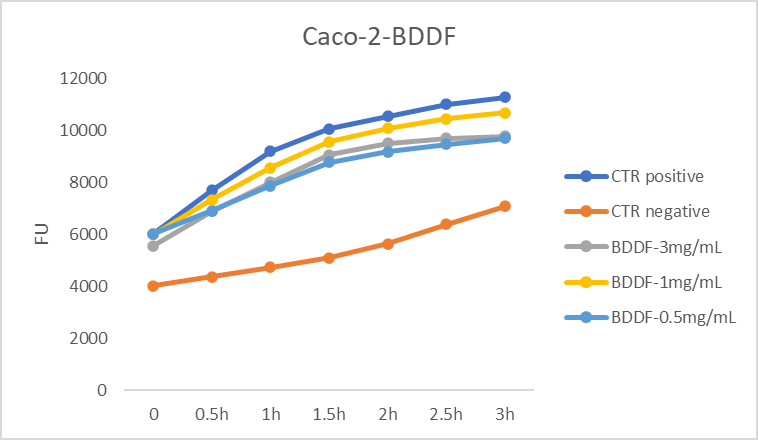

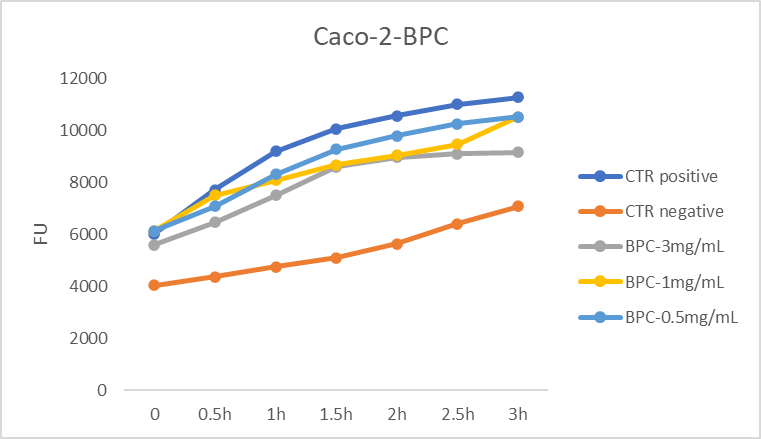


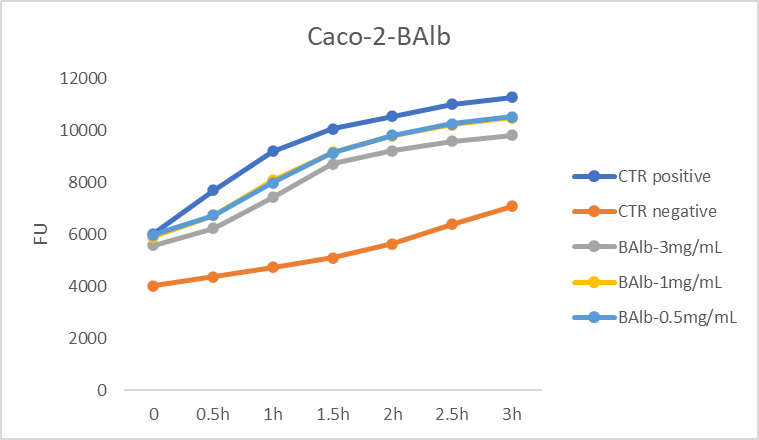

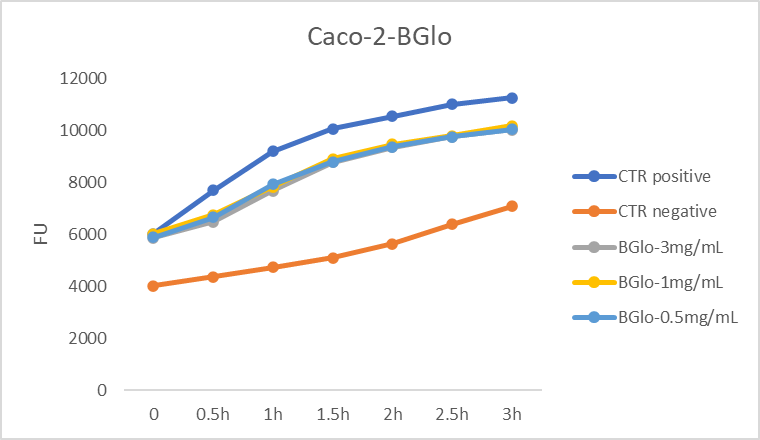


**Figure 3S**. Kinetic evaluation of ROS production in Caco-2 cells and RAW264.7 macrophages in the presence of Mexican and British chia samples. **A)** ROS of Mexican chia samples in RAW264.7 Macrophages, **B)** ROS of British chia samples in RAW264.7 Macrophages, **C)** ROS of Mexican chia samples in Caco-2 cells **D)** ROS of British chia samples in Caco-2 cells. Abbreviations: DDF, degummed-defatted chia flour; PC, protein concentrate; Alb, albumin; Glo, globulin; M, Mexican chia samples; B, British chia samples.

**Table 1S**. Peptide molecular weight distribution of chia (*Salvia hispanica* L.) samples and digestates.

| **Peptide molecular weight distribution %** | | | |
| --- | --- | --- | --- |
| **Sample** | **U** | **GP** | **IP** |
| **< 0.2 kDa** | | | |
| MDDF | 12.09 ± 0.41^c,A^ | 4.31 ± 0.18^c,B^ | 10.44 ± 0.01^cd,A^ |
| BDDF | 11.27 ± 0.16^c,A^ | 3.42 ± 0.25^c,B^ | 10.37 ± 0.18^cd,A^ |
| MPC | 6.54 ± 0.79^d,A^ | 3.61 ± 0.02^c,B^ | 8.32 ± 0.07^d,A^ |
| BPC | 4.62 ± 0.21^de,A^ | 2.02 ± 0.03^c,B^ | 8.18 ± 0.28^d,A^ |
| MAlb | 31.52 ± 2.45^a,A^ | 25.15 ± 0.71^a,B^ | 21.90 ± 1.75^a,C^ |
| BAlb | 27.82 ± 0.12^b,A^ | 18.09 ± 0.18^b,B^ | 17.45 ± 0.57^b,B^ |
| MGlo | 3.48 ± 0.55^e,B^ | 3.01 ± 0.39^c,B^ | 11.44 ± 0.24^c,A^ |
| BGlo | 4.11 ± 0.04^de,B^ | 2.27 ± 0.51^c,B^ | 11.46 ± 0.02^c,A^ |
| **0.2-0.5 kDa** | | | |
| MDDF | 13.75 ± 1.75^a,B^ | 9.11 ± 0.04^ab,C^ | 25.55 ± 0.15^a,A^ |
| BDDF | 10.81 ± 1.60^ab,B^ | 4.84 ± 0.21^b,C^ | 25.91 ± 0.47^a,A^ |
| MPC | 10.86 ± 1.02^ab,B^ | 3.92 ± 0.08^b,C^ | 22.80 ± 0.12^a,A^ |
| BPC | 2.35 ± 0.70^c,B^ | 1.88 ± 1.10^b,B^ | 22.37 ± 0.60^a,A^ |
| MAlb | 11.32 ± 1.50^ab,B^ | 11.74 ± 1.97^a,B^ | 16.00 ± 0.04^b,A^ |
| BAlb | 10.82 ± 0.06^ab,A^ | 7.09 ± 0.16^ab,B^ | 12.54 ± 0.13^c,A^ |
| MGlo | 13.45 ± 0.39^a,A^ | 2.38 ± 1.15^b,B^ | 13.13 ± 0.78^bc,A^ |
| BGlo | 8.05 ± 0.06^b,B^ | 1.23 ± 0.01^b,C^ | 13.72 ± 0.02^bc,A^ |
| **0.5-1 kDa** | | | |
| MDDF | 75.61 ± 0.71^a,A^ | 18.79 ± 0.30^c,C^ | 64.02 ± 0.14^b,B^ |
| BDDF | 63.08 ± 0.83^b,A^ | 17.76 ± 0.34^c,B^ | 63.72 ± 0.30^b,A^ |
| MPC | 58.39 ± 2.15^c,A^ | 16.80 ± 0.54^cd,B^ | 44.10 ± 0.75^d,A^ |
| BPC | 42.81 ± 0.56^e,A^ | 16.89 ± 0.81c^,B^ | 42.14 ± 0.44^d,A^ |
| MAlb | 48.56 ± 0.74^d,A^ | 43.30 ± 0.43^a,A^ | 47.98 ± 2.95^d,A^ |
| BAlb | 50.05 ± 0.69^d,A^ | 34.67 ± 1.57^b,B^ | 54.55 ± 1.32^c,A^ |
| MGlo | 60.90 ± 0.64^bc,B^ | 21.58 ± 0.28^c,C^ | 75.44 ± 1.03^a,A^ |
| BGlo | 61.90 ± 0.08^bc,B^ | 12.01 ± 0.15^d,C^ | 74.83 ± 0.01^a,A^ |
| **>1 kDa** | | | |
| MDDF | - | 69.52 ± 2.53^c^ | - |
| BDDF | 16.24 ± 2.59^c,B^ | 73.99 ± 0.29^c,A^ | - |
| MPC | 24.22 ± 0.33^b,B^ | 75.69 ± 0.64^bc,A^ | 24.79 ± 0.70^a,B^ |
| BPC | 50.24 ±0.35^a,B^ | 79.22 ± 1.94^b,A^ | 27.88 ± 0.35^a,C^ |
| MAlb | 8.54 ± 1.59^d,C^ | 21.32 ± 1.84^e,A^ | 15.63 ± 0.28^b,B^ |
| BAlb | 11.33 ±0.88^d,C^ | 40.23 ± 1.48^d,A^ | 16.06 ± 0.45^b,B^ |
| MGlo | 22.18 ± 0.81^b,B^ | 72.85 ± 2.11^c,A^ | - |
| BGlo | 25.94 ± 0.18^b,B^ | 84.50 ± 0.67^a,A^ | - |

Data are the mean ± SD of the replicates. Different lowercase letter within the column indicates statistical differences among samples at the same digestion phase (*p* < 0.05, Tukey test). Different uppercase letter within the row indicates statistical differences among the same chia samples at different digestion phase (*p* < 0.05, Tukey test). Abbreviations: MDDF, Mexican degummed-defatted flour; BDDF, British degummed-defatted flour; MPC, Mexican protein concentrates; BPC, British protein concentrates; MAlb, Mexican chia albumin; BAlb, British chia albumin; MGlo, Mexican chia globulin; BGlo, British chia globulin; U, undigested sample; G, gastric digestion endpoint; I, intestinal digestion endpoint.
